# Genetic Regulation of Vertebrate Forebrain Development by Homeobox Genes

**DOI:** 10.3389/fnins.2022.843794

**Published:** 2022-04-25

**Authors:** Ryan F. Leung, Ankita M. George, Enola M. Roussel, Maree C. Faux, Jeffrey T. Wigle, David D. Eisenstat

**Affiliations:** ^1^Murdoch Children’s Research Institute, The Royal Children’s Hospital Melbourne, Parkville, VIC, Australia; ^2^Department of Paediatrics, University of Melbourne, Parkville, VIC, Australia; ^3^Department of Surgery, Royal Melbourne Hospital, The University of Melbourne, Parkville, VIC, Australia; ^4^Department of Biochemistry and Medical Genetics, Max Rady College of Medicine, Rady Faculty of Health Sciences, University of Manitoba, Winnipeg, MB, Canada; ^5^Institute of Cardiovascular Sciences, St. Boniface Hospital Albrechtsen Research Centre, Winnipeg, MB, Canada; ^6^Department of Medical Genetics, University of Alberta, Edmonton, AB, Canada; ^7^Department of Pediatrics, University of Alberta, Edmonton, AB, Canada; ^8^Department of Oncology, Faculty of Medicine & Dentistry, University of Alberta, Edmonton, AB, Canada

**Keywords:** forebrain, development, homeobox, bHLH factor, forkhead (Fkh) transcription factors, DNA binding domain

## Abstract

Forebrain development in vertebrates is regulated by transcription factors encoded by homeobox, bHLH and forkhead gene families throughout the progressive and overlapping stages of neural induction and patterning, regional specification and generation of neurons and glia from central nervous system (CNS) progenitor cells. Moreover, cell fate decisions, differentiation and migration of these committed CNS progenitors are controlled by the gene regulatory networks that are regulated by various homeodomain-containing transcription factors, including but not limited to those of the *Pax* (paired), *Nkx*, *Otx* (orthodenticle), *Gsx/Gsh* (genetic screened), and *Dlx* (distal-less) homeobox gene families. This comprehensive review outlines the integral role of key homeobox transcription factors and their target genes on forebrain development, focused primarily on the telencephalon. Furthermore, links of these transcription factors to human diseases, such as neurodevelopmental disorders and brain tumors are provided.

## Introduction

### Overview of Forebrain Development

Early brain development is marked by the formation of different compartments through the segmentation of the neural tube that is guided and defined by specific regional expression of transcription factors. The developing brain is sectioned into three contiguous parts, the prosencephalon in the most anterior area, which then matures into the forebrain; the mesencephalon following posteriorly, which give rises to the midbrain; and further posteriorly the rhombencephalon, the early form of the hindbrain. These areas further partition, where the prosencephalon separates into primary prosencephalon (diencephalon) and secondary prosencephalon (telencephalon) (Puelles, 2013, 2018), and the rhombencephalon divides into the metencephalon and myelencephalon. In contrast to the other two regions, the mesencephalon does not divide (Stiles, 2008). Within the forebrain, the prosomeric model depicts the division of this area into 7 segments called the prosomeres (Rubenstein et al., 1994; Puelles and Rubenstein, 2003). The diencephalon develops into 3 prosomeres (p1, p2, p3), which are then recognized as the pretectum, thalamus and pre-thalamus. The secondary prosencephalon develops into two hypothalamo-telencephalic prosomeres (hp1, hp2), later giving rise to the hypothalamus and telencephalon. The mesencephalon contributes to two prosomeres (m1, m2) (Puelles, 2018).

**FIGURE 1:**
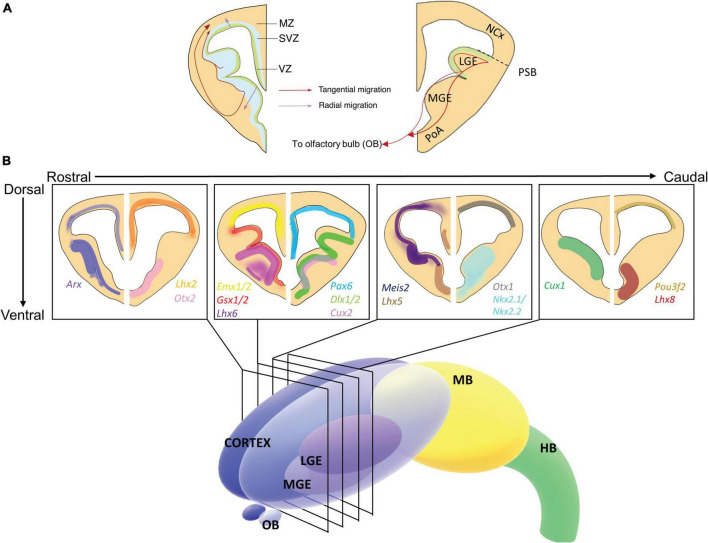
Expression of homeobox genes in the developing embryonic mouse forebrain. **(A)** Schematic illustration of coronal section of E13.5 forebrain depicting ventricular zone (VZ), subventricular zone (SVZ), and mantle zone (MZ) on the left-hand side and neocortex (NCx), lateral ganglionic eminence (LGE), and medial ganglionic eminence (MGE) on the right-hand side. The VZ and SVZ are the proliferative zones, comprised of progenitor cells. Depending on the identity of these differentiated cells, the cells migrate either tangentially (red arrows) or radially (purple arrows) into the MZ and proceed to mature (Left-hand side). Migration toward the olfactory bulb from the VZ of the LGE also occurs (Right-hand side). **(B)** 3-dimensional schematic of the developing forebrain. The LGE and MGE are contained within the cortex, above the olfactory bulbs (OB). The midbrain (MB) and hindbrain (HB) are also labeled. Insets show schematic representations of 4 coronal sections taken from the forebrain depicting the expression of key homeobox gene expression patterns from rostral to caudal at embryonic time point E13.5. Gene name colors correspond to the expression color shown in the section. Transcription factor expression can be overlapping or structurally distinct and is related to the function of the individual transcription factor (Allen Institute for Brain Science, 2019). *Arx* and *Meis2*, to an extent, are expressed throughout the forebrain, whereas *Lhx2, Emx1/2, Pax6, Otx1*, and *Pou3f2* are expressed in the neocortex and pallium. *Dlx1/2, Gsx1, Otx2*, and *Cux1* are expressed in the GE, *Gsx2* is expressed specifically in the LGE, and *Nkx2.1, Cux2, Lhx6*, and *Lhx8* in the MGE. *Irx3* is not depicted here as it is expressed in the thalamus (not shown). For detailed depictions of gene expression patterns, readers are encouraged to review the cited primary references or the Allen Brain Atlas: Developing Mouse Brain (Allen Institute for Brain Science, 2019). NCx, neocortex; LGE, lateral ganglionic eminence; MGE, medial ganglionic eminence; V, ventricle; VZ, ventricular zone; SVZ, subventricular zone; MZ, mantle zone.

The regions adjacent to the ventricular surface in the brain are the ventricular zone (VZ), followed by the subventricular zone (SVZ), and the mantle zone (MZ) (Figure 1A). The VZ contains radial glia, which then differentiate into intermediate neural progenitors that populate the SVZ, where both of these cell types can give rise to neurons (Miyata et al., 2001; Noctor et al., 2001, 2004; Haubensak et al., 2004). The telencephalon can be divided into the dorsal (pallium) and ventral (subpallium) telencephalon, where the neocortex and the ganglionic eminences (GE) are located, respectively. The anatomic region separating the dorsal and ventral telencephalon is often referred to as the pallio-subpallial boundary (PSB). The GE is divided into lateral, medial, and caudal GE (LGE; MGE; CGE), and ventral to the MGE is the preoptic area (PoA) (Figure 1A). The LGE can be further separated in the ventral LGE (vLGE), where striatal projection neurons originate, and the dorsal LGE (dLGE) that gives rise to intercalated cells of the amygdala and neurons in the olfactory bulb along with the lateral LGE wall (Yun et al., 2001; Stenman et al., 2003; Waclaw et al., 2010). The LGE is a local source of retinoic acid, a morphogen that regulates cortical patterning and regionalization (see Shibata et al., 2021; Ziffra et al., 2021 for more details) (Toresson et al., 1999; Molotkova et al., 2007; Shibata et al., 2021; Ziffra et al., 2021).

### Origin of Cortical and Striatal Neurons

Excitatory and inhibitory neuronal activities need to be balanced in order for the nervous system to maintain homeostasis and to optimally process information; these are governed by projection and inhibitory neurons in the brain, respectively. Neuronal progenitor cells (NPC) are produced in both dorsal and ventral telencephalon; NPCs from the dorsal telencephalon give rise to projection neurons (glutamatergic) and NPCs from the ventral telencephalon differentiate into inhibitory interneurons (γ-amino butyric (GABA)-ergic) (Anderson et al., 1997a, 2002b). These neuronal origin sites are conserved amongst mammals, as shown through studies in primates, rodents, and humans, in which some cortical interneurons could be generated locally in the dorsal telencephalon (Letinic et al., 2002; Hansen et al., 2013; Ma et al., 2013). Glutamatergic neurons make up ∼ 70% of the neuronal population in the mouse, with the remaining ∼ 30% being GABAergic interneurons (Hendry et al., 1987). Within the ventral telencephalon, GABAergic interneurons are produced mainly from *Nkx2.1* expressing progenitor cells in the MGE and PoA (Fogarty et al., 2007; Gelman et al., 2009), and migrate tangentially to reach the neocortex (Marín and Rubenstein, 2003). These ventral telencephalic interneurons mainly consist of parvalbumin (*pva*^+^), somatostatin (*sst*^+^), and *5ht3a+* interneurons subtypes (Rudy et al., 2011). Many *sst+* interneurons arise and migrate from the CGE, while other interneuron subtypes arise from progenitor cells in the LGE and CGE, including the vasoactive intestinal peptide and cholecystokinin expressing interneurons which reside in the MZ (Anderson et al., 2001; Nery et al., 2002; Miyoshi et al., 2010). The main population of striatal projection neurons comprises the GABAergic medium spiny neurons (MSNs) which arise from progenitors in the LGE, and account for ∼ 80% of the striatal neuron population in primates and rodents (Graveland and DiFiglia, 1985). Some key marker genes for MSN differentiation include *Foxp1/2*, *Ascl1*, *Ebf1*, and *Meis2* (Garel et al., 1999; Toresson et al., 1999; Carri et al., 2013). The differentiation of MSNs is dependent on the temporal expression of a set of transcription factors, particularly the repressive function of *Dlx1/2* on *Ascl1* at specific timepoints, to promote differentiation and migration of striatal neurons (Anderson et al., 1997b; Yun et al., 2002). EBF1 then controls later differentiation and migration from the SVZ to the MZ (Garel et al., 1999).

### Olfactory Bulb Neurogenesis

In mice, olfactory bulb neurogenesis occurs from embryonic until early postnatal stages, and is dependent on the neuronal types (Alvarez-Buylla and Lim, 2004; Tucker et al., 2006; Figueres-Oñate and López-Mascaraque, 2016). Initially, projection neurons are generated by E12.5, followed by the development of inhibitory interneurons by E14.5 (Bayer, 1983; Tucker et al., 2006). The olfactory bulb projection neurons, mitral/tufted (M/T) cells, originate from progenitor cells in the pallium and are differentiated from *Pax6*+ radial glia (Whitman and Greer, 2009; Imamura and Greer, 2013). M/T cells can adopt both radial and tangential migration. Earlier born neurons predominantly migrate radially and populate the deeper cortical layers, while later born projection neurons are more likely to migrate tangentially to the superficial cortical layer (Imamura et al., 2011). Migration of these projection neurons is regulated by a number of transcription factors, such as PAX6 and LHX2, which are also crucial for cortical neuron migration (Nomura et al., 2007; Saha et al., 2007). Transcription factors specific for olfactory bulb projection neuron migration include *Ap2-epsilon*, *Arx*, and *FezF1*, which are all important for proper orientation of M/T cells, as well as the expression of *Tbr1/2* (Yoshihara et al., 2005; Feng et al., 2009; Shimizu and Hibi, 2009; Imamura and Greer, 2013).

Olfactory bulb interneurons, in contrast to cortical interneurons, are derived from the dLGE, and postnatally in the SVZ, with the exception of *Emx1+* pallial progenitors (Wichterle et al., 2001; Stenman et al., 2003). Subsequently, these interneurons tangentially migrate through to the olfactory bulb, postnatally through the rostral migratory stream (Kriegstein and Alvarez-Buylla, 2009). Although born in neuroanatomic regions distinct from cortical interneurons, olfactory bulb interneuron migration is regulated by a similar set of factors. Some of these include *Dlx1/2*, *Ascl1*, and *Robo-Slit* (Andrews et al., 2006; Long et al., 2007). Upon reaching the olfactory bulb, the interneurons differentiate into GABAergic interneurons and subsequently, subtype specification takes place (Lois and Alvarez-Buylla, 1994; Sequerra, 2014) which is itself dependent on the developmental stage, i.e., whether born at an embryonic or postnatal stage (De Marchis et al., 2007; Batista-Brito et al., 2008). Examples of transcription factors that regulate interneuron development are *Sp8/Sp9* which are essential for olfactory bulb development (Li et al., 2017). For a more in-depth discussion about olfactory bulb development refer to a recent review from Tufo et al. (2022).

### Radial and Tangential Migration of Neurons

There are two modes of neuronal migration, radial and tangential, classified by the axis of migration (Figure 1B). Cells move from the VZ toward the MZ generally by radial migration, and can descend within the VZ before migrating toward the MZ. *Radial migration* occurs during the development of the cerebral cortex, spinal cord, striatum and thalamus (Ayala et al., 2007). Morphological changes of interneurons mark the start of radial migration, whereas restriction of such changes also impairs the migration of these interneurons (LoTurco and Bai, 2006). Two different modes of movements are adopted during radial migration (Nadarajah et al., 2001). Interneurons migrate by somal translocation, by attaching to the outer surface of the developing brain (pial surface) and as microtubules shorten, the nucleus is pulled forward (Franco et al., 2011). Locomotion, on the other hand, allows interneurons to be guided by radial glial cells toward the destination during the radial migration through complex forebrain structures (Rakic, 1972).

*Tangential migration* is adopted by cortical interneurons born in the GE, as these cells need to migrate from the GE to the neocortex while avoiding movement toward the striatum (DeDiego et al., 1994). Despite being derived in different areas, interneurons arising from the MGE, CGE and preoptic area have a similar transcriptome (Mayer et al., 2018), which could contribute to the similar migration pattern these interneurons adopt. Transcription factors tightly regulate the migration fate of interneurons, such as the expression or repression of *Nkx2.1* determines whether interneurons migrate into the striatum or neocortex, respectively (Nóbrega-Pereira et al., 2008). There are two major paths for interneurons to migrate from the GE to the developing neocortex, through a superficial route that bypasses the MZ or a deeper route that passes through the SVZ (Figure 1A; Wichterle et al., 2001). These migration paths are guided by signaling molecules such as the chemokine CXCL12, which attract interneurons, and its receptor CXCR4. Studies have shown that disruption of CXCL12 or its receptor CXCR4 led to interneuronal mislocalization (Stumm et al., 2003; López-Bendito et al., 2008; Wang et al., 2011b). Furthermore, *Tbr2*+ cortical intermediate progenitor cells may actively attract interneuron migration into the cortex, which is concurrently modulated by CXCL12 signaling (Sessa et al., 2010). Another chemokine, Neuregulin 3 (*Nrg3*), mediated by ErbB4 attracts and regulates the final destination of GABAergic interneurons in the cortex (Rakić et al., 2015). Similarly, repulsive guidance cues Semaphorin 3A and 3F also play a role in guiding interneuron tangential migration, where their expression in the LGE prevents interneuron migration toward the basal area (Chen et al., 2008). This repulsion is achieved by the interactions between these molecules and their receptors neuropilin-1 (*Nrp1*) and neuropilin-2 (*Nrp2*), which are expressed in migrating interneurons (Marín et al., 2001). Some other extrinsic factors act as mitogens to provide motility and control the rate of migration for interneurons, such as the hepatocyte growth factor/scatter factor (Powell et al., 2001). Furthermore, GABA itself can act as a motogen and accelerate tangential migration (Inada et al., 2011). These processes that direct neuron fate determination are ultimately regulated by members of the homeobox and basic helix-loop-helix (bHLH) transcription factor families (Table 1).

**TABLE 1:** Summary of selected transcription factors required for forebrain development.

Gene family	Gene symbol	Human chromosome location^#^	Forebrain expression at E13.5	Forebrain gene function
Homeobox	*Arx*	Xp22.13	Cortex VZ; GE SVZ (Miura et al., 1997)	Promotes GABAergic interneuron tangential migration (Friocourt and Parnavelas, 2010; Olivetti and Noebels, 2012)
	*Cux1*	7q22.1	GE VZ and SVZ (Nieto et al., 2004; Zimmer et al., 2004)	Represses dendritic arborization (Coqueret et al., 1998)
	*Cux2*	12q24.11-q24.12	MGE SVZ	Controls neuronal specification and differentiation in the upper cortical layers (Zimmer et al., 2004)
	*Dlx1*	2q33.1 (Stock et al., 1996)	GE VZ and SVZ (Pleasure et al., 2000)	Regulates GABAergic interneuron specification and migration (Simeone et al., 1992; Anderson et al., 1997a,b)
	*Dlx2*	2q31.1 (Stock et al., 1996)	GE VZ and SVZ (Pleasure et al., 2000)	Regulates GABAergic interneuron specification and migration (Anderson et al., 1997a,b)
	*Emx1*	2p13.2	Cortex VZ (Simeone et al., 1992; Yoshida et al., 1997)	Dorsal forebrain specification and patterning (Yoshida et al., 1997; Stocker and O’Leary, 2016)
	*Emx2*	10q26.11	Cortex VZ and SVZ (Simeone et al., 1992; Yoshida et al., 1997)	Dorsal forebrain specification and patterning (Yoshida et al., 1997; Hamasaki et al., 2004)
	*Gsx1*	13q12.2	dLGE VZ (Toresson and Campbell, 2001)	Promote OPC proliferation (Chapman et al., 2018)
	*Gsx2*	4q12	vLGE VZ (Yun et al., 2001)	Promote neuron, oligodendrocyte, and glia specification (Kessaris et al., 2006; Fogarty et al., 2007; Chapman et al., 2018)
	*Irx3*	16q12.2	Thalamus (Robertshaw et al., 2013)	Promotes differentiation in the thalamus and neurogenesis at the paraventricular nucleus of the hypothalamus (Robertshaw et al., 2013; Smemo et al., 2014)
	*Lhx2*	9q33.3	Cortex VZ and SVZ (Roy et al., 2014)	Progenitor cell proliferation; dorsal patterning (Godbole et al., 2018)
	*Lhx5*	12q24.13	Ventral forebrain (Sheng et al., 1997)	Hippocampal neuron differentiation and migration (Abellán et al., 2010)
	*Lhx6*	9q33.2	MGE SVZ (Matsumoto et al., 1996)	Regulates GABAergic interneuron differentiation and migration (Alifragis et al., 2004; Zhao et al., 2008; Neves et al., 2013)
	*Lhx8*	1p31.1	MGE MZ (Matsumoto et al., 1996)	Regulates cholinergic interneuron differentiation and specification (Zhao et al., 2003; Fragkouli et al., 2005; Lopes et al., 2012)
	*Meis2*	15q14	Cortex VZ; LGE, MGE, and CGE (Cecconi et al., 1997; Toresson et al., 1999; Agoston et al., 2014)	Controls gene expression and promotes differentiation and migration of neurons (Agoston et al., 2014)
	*Nkx2.1*	14q13.3	MGE and PoA (Xu et al., 2005)	Ventral forebrain specification and patterning (Nóbrega-Pereira et al., 2008; Kanatani et al., 2015)
	*Nkx2.2*	20p11.22	MGE (Ericson et al., 1997)	Promotes GABAergic interneuron specification (Briscoe et al., 1999; Robertshaw et al., 2013)
	*Otx1*	2p13 (Kastury et al., 1994)	Cortex VZ (Hoch et al., 2015)	Dorsal forebrain specification and patterning (Larsen et al., 2010b)
	*Otx2*	14q21-22 (Kastury et al., 1994)	GE VZ (Hoch et al., 2015)	Ventral forebrain specification and patterning (Larsen et al., 2010b)
	*Pax6*	11q13	Cortex VZ (Hirata et al., 2002)	Dorsal forebrain specification and patterning (Scardigli et al., 2003)
	*Pou3f2*	6q16.1	Cortex VZ (Nakai et al., 1995; Dominguez et al., 2012)	Regulates neuronal differentiation and radial migration in the telencephalon (Artavanis-Tsakonas et al., 1999)
bHLH	*Ascl1*	12q23.2	GE VZ (Fode et al., 2000; Britz et al., 2006)	Interneuron specification from neural progenitor cells (Nieto et al., 2001; Bertrand et al., 2002)
	*Olig1*	21q22	GE VZ and SVZ (Takebayashi et al., 2000)	Promotes oligodendrocyte differentiation and specification (Tekki-Kessaris et al., 2001; Anderson et al., 2002a; Lu et al., 2002)
	*Olig2*	21q22	GE VZ and SVZ (Takebayashi et al., 2000)	Promotes oligodendrocyte differentiation and interneuron specification (Tekki-Kessaris et al., 2001; Anderson et al., 2002a; Lu et al., 2002)
	*Olig3*	6q24	Dorsal thalamus (Takebayashi et al., 2000)	Promotes interneuron specification (Takebayashi et al., 2002; Lowenstein et al., 2021)
Forkhead	*Foxg1*	14q12	Ventral forebrain (Tao and Lai, 1992)	Ventral forebrain specification; regulates neuron migration and specification (Martynoga et al., 2005; Kumamoto and Hanashima, 2017)

*^#^According to NCBI database (NCBI Datasets, 2021).*

### Homeobox Genes

**FIGURE 2:**
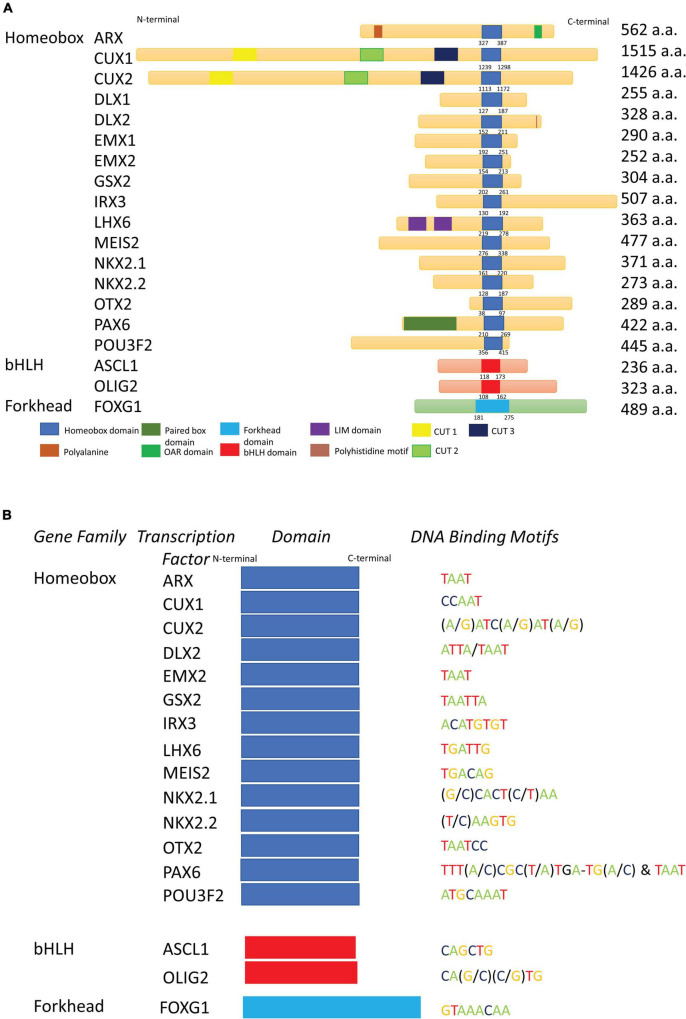
Homeobox transcription factors and their key functional domains. **(A)** Schematic depiction of the domain structure of selected homeobox, bHLH and forkhead transcription factors illustrating the highly conserved nature of the homeodomains and other DNA-binding domains of these transcription factors. Other significant functional domains are also shown. DLX2, but not DLX1, contains a polyhistidine motif in its C-terminus. PAX6 contains both a paired domain, as well as the HD. ARX, along with a HD, also contains a polyalanine repeat and an Aristaless domain. a.a., amino acid. **(B)** The DNA binding sites for Homeobox, bHLH and Forkhead transcription factors from **(A)**. A consensus DNA binding motif for DLX1 and EMX1 is not available.

Homeobox genes are an important gene family for embryonic development, defined by a conserved homeodomain (HD) containing a helix-loop-helix-turn-helix structure (Gehring et al., 1994; Noyes et al., 2008). The 60 amino acid HD is commonly located at the carboxyl terminal end of the protein, and binds DNA primarily through the 50th residue, usually being a glutamine, allowing homeobox genes to function as transcription factors (Figure 2; Kappen, 2000). This DNA binding motif is located in the second and third helices, which recognizes and binds to the major groove of DNA at specified consensus sites (Table 2). Further, the N-terminal arm contributes to the binding strength through interactions with the DNA minor groove, typically through a basic residue such as arginine at the 5^th^ residue in the HD (Rohs et al., 2009). Apart from the consensus binding sequence, other important factors for DNA binding specificity include cofactors and additional DNA binding domains, such as the paired domain (PRD) in PAX superfamily members. Water molecules have been shown to be crucial for the HD to bind DNA (Billeter et al., 1996). Protein-protein interactions driven by the flanking regions around HD also increase the specificity of DNA binding (Li et al., 1995; Amin et al., 2015; Merabet and Lohmann, 2015). Homeobox proteins often contain other domains apart from the HD, which provide additional DNA specificity for these proteins, and have allowed characterization of homeobox proteins into 11 different classes, such as the Antennapedia (ANTP), Paired (PRD), LIM and NK classes (Holland et al., 2007), and can be further divided into different families within these classes. Large functional and comparative genomics studies have enabled analyses of these proteins, and allowed accurate annotation, naming and classification of homeobox genes (Holland et al., 2007).

**TABLE 2:** DNA binding motifs and selected target genes.

Gene family	Gene symbol	DNA binding motif	Target genes
Homeobox	*Arx*	TAAT (Cho et al., 2012)	*Cxcr4; Cxcr7; Dlx2; Ebf3; Lhx8* (Fulp et al., 2008; Colasante et al., 2009; Quille et al., 2011)
	*Cux1*	CCAAT (Moon et al., 2000)	*Nfib; Fesf2; Pou6f2; Sox5* (Gray et al., 2017)
	*Cux2*	(A/G)ATCAAT (Conforto et al., 2012)	*Xlr3b; Xlr4b* (Cubelos et al., 2010)
	*Dlx2*	ATTA/TAAT (Zhou et al., 2004)	*Dlx5/6; Gad1/2; Gsx2; Lhx6/8; Nrp2; Olig2; Otx2; Pax6* (Petryniak et al., 2007; Long et al., 2009; Lindtner et al., 2019)
	*Emx1*		*Nrp1* (Lim et al., 2015)
	*Emx2*	TAAT (Beckmann et al., 2011)	*Gsx2; Sox2; Ten-1* (Mariani et al., 2012; Desmaris et al., 2018)
	*Gsx2*	TAATTA (Salomone et al., 2021)	*Dbx1; Dlx1/*2 (Corbin et al., 2000; Toresson et al., 2000; Yun et al., 2001)
	*Irx3*	ACATGTGT (Bilioni et al., 2005)	*Sox14; Gbx2* (Robertshaw et al., 2013; Smemo et al., 2014)
	*Lhx8*	TGATTG (Park et al., 2012)	*Lhx6; Shh* (Zhao et al., 2003; Flandin et al., 2011)
	*Meis2*	TGACAG (Chang et al., 1997)	*Dlx1/2; Dlx5/6* (Ghanem et al., 2003)
	*Nkx2.1*	(G/C)CACT(C/T)AA (Manoli and Driever, 2014)	*Gbx1/2; Gli2; Lhx6/8; Pax6; Nrp1/2* (Nóbrega-Pereira et al., 2008; Kanatani et al., 2015; Sandberg et al., 2016)
	*Otx2*	TAATCC/T (Briata et al., 1999)	*Arx; Dbx1; Dlx1/2; Fgf8; Hes1; Nkx2.1; Olig1/2; Pax3; Ten-*C (Gherzi et al., 1997; Hoch et al., 2015)
	*Pax6*	TTT(A/C)CGC(T/A)TGA-TG(A/C) and TAAT (Sun et al., 2015)	*Ascl1; Dlx2; Emx1/2; Ngn2; Pax6* (Scardigli et al., 2003; Sun et al., 2015)
	*Pou3f2*	ATGCAAAT (Herr and Cleary, 1995)	*Ascl1; Trim8; Vrk2* (Artavanis-Tsakonas et al., 1999; Chen et al., 2018; Pearl et al., 2019)
bHLH	*Ascl1*	CAGCTG (Webb et al., 2013)	*Ccng2; Cdk1/2Dlx2; EphB2; E2f1; Gadd45g; Hipk2; NeuroD; Ngn1* (Cau et al., 2002; Webb et al., 2013; Park et al., 2017)
	*Olig1*	CA(G/A)NTG (Li et al., 2007)	*Dlx1/2* (Silbereis et al., 2014)
	*Olig2*	CA(G/C) (C/G)TG (Küspert et al., 2011)	*Irx3; Ngn2; Nkx2.2; Pax6; Sox10; Zep2* (Küspert et al., 2011; Emery and Lu, 2015)
Forkhead	*Foxg1*	GTAAACAA (Dai et al., 2020)	*Ascl1; Cxcr4/7; Ccnd1; Dlx1/2; Eph44; Fgf8; Prox1; Robo1; Sema3A/F* (Bulstrode et al., 2017; Yang et al., 2017; Hou et al., 2019)

### Basic Helix-Loop-Helix Genes

Basic helix-loop-helix (bHLH) proteins are another superfamily of transcription factors present in most eukaryotes, with critical functions during embryonic development, such as neurogenesis and myogenesis. bHLH domains contains two alpha helices, helix 1 and helix 2. These helices are connected by a short loop, and at the amino-terminal end of helix 1 is a basic region (Murre et al., 1989). This basic region binds DNA by recognizing a core CANNTGG motif, known as an *E-box motif*, and is specific for different transcription factors (Table 2). Upon binding, the basic region is fitted into the major grove of the DNA. The HLH domain interacts with other proteins, forming different homo- or hetero-dimeric complexes that are required for DNA binding (Ellenberger et al., 1994). The unique combinations of these bindings give rise to the diverse transcriptional regulatory functions of bHLH proteins during development. bHLH proteins can be roughly divided into those that are either cell-type specific or widely expressed where the group of transcription factors governing neuron development are often referred to as proneural proteins (Lee, 1997; Srivastava et al., 1997).

## Selected Transcription Factors Encoded by Homeobox Genes

In the following major section of this comprehensive review, detailed summaries of 21 homeobox genes (in alphabetical order) that encode homeodomain containing transcription factors are provided. These genes were selected due to their essential role in forebrain development. However, we acknowledge that this selection of genes excludes several other important homeobox genes as well as key bHLH (*Ascl1*, *Olig1*, *Olig2*, and *Olig3*) and forkhead (*Foxg1*) genes required for neurodevelopment. For this reason, we have included *Ascl1*, *Olig1/2/3*, and *Foxg1* in Figure 2 and the Tables.

A brief note about gene and protein nomenclature is useful. By consensus: mouse gene, *Dlx*; zebrafish gene, *dlx*; human gene, *DLX*; mouse and human protein, DLX.

### Aristaless Related Homeobox Gene

The Aristaless related homeobox (*Arx*) paired-like HD transcription factor is the vertebrate homolog of the *Drosophila* aristaless (*al*) gene, which is essential for appendage formation (Miura et al., 1997). The gene is located on human chromosome Xp22.13 and is reported to be involved in neurological disorders such as X-linked intellectual disabilities (Table 3; Bienvenu et al., 2002; Friocourt and Parnavelas, 2010). In vertebrate embryogenesis, *Arx* transcriptionally regulates interneuron specification and migration (Fulp et al., 2008; Friocourt and Parnavelas, 2010; Olivetti and Noebels, 2012). ARX contains multiple structural domains and motifs, including the HD, a PRD-like domain, an N-terminal octapeptide domain, a central acidic domain and the C-terminal aristaless domain as well as three nuclear localization sequences and four polyalanine (polyA) tracts (Miura et al., 1997; Figure 2). ARX binds the transcriptional co-repressor TLE1, through the TLE1 octapeptide domain, and recognizes DNA at TAAT sites (Jennings et al., 2006; McKenzie et al., 2007; Cho et al., 2012). *In vitro* assays show that although ARX can be phosphorylated at multiple sites, it is unclear whether ARX functions are regulated by its phosphorylation state (Mattiske et al., 2018; Shi et al., 2020).

**TABLE 3:** Forebrain mutant phenotypes and related diseases.

Gene family	Gene symbol	Forebrain mutant phenotype description	Related neural diseases
Homeobox	*Arx*	Disrupted GABAergic interneuron migration; structural brain malformation (Kitamura et al., 2002; Colombo et al., 2007)	Epilepsy; X-linked lissencephaly or intellectual disability (Kato et al., 2004; Friocourt et al., 2008)
	*Dlx1/2*	Disrupted GABAergic interneuron differentiation and migration (Anderson et al., 1997a,b; MacDonald et al., 2013)	Down Syndrome; epilepsy; Rett Syndrome; schizophrenia (Cobos et al., 2005b; Poitras et al., 2007)
	*Emx1*	Absence of corpus callosum; postnatal cKO disrupted cortex patterning (Qiu et al., 1996; Stocker and O’Leary, 2016)	Tumor suppressor for glioblastoma (Jimenez-García et al., 2021b)
	*Emx2*	Reduced cortex size; impaired radial migration (Falcone et al., 2016; Monnier et al., 2018)	Tumor suppressor for glioblastoma (Jimenez-García et al., 2021a)
	*Gsx2*	Reduced LGE size; reduced amount of GABAergic interneurons (Yun et al., 2003)	Basal ganglia malformation; Parkinson’s Disease; Huntington’s Disease (Zuccoli et al., 2015; De Mori et al., 2019)
	*Lhx5*	Impaired hippocampus formation (Abellán et al., 2010)	
	*Lhx6*	Reduced GABAergic interneuron subtype amount; disrupted interneuron migration (Liodis et al., 2007; Neves et al., 2013)	Tourette Syndrome; schizophrenia (Volk et al., 2014; Donegan et al., 2020; Pagliaroli et al., 2020)
	*Lhx8*	Impaired interneuron differentiation (Manabe et al., 2007, 2008)	Tourette Syndrome (Pagliaroli et al., 2020)
	*Nkx2.1*	Increased amount of GABAergic interneuron (Sussel et al., 1999; Fragkouli et al., 2009)	Schizophrenia; impaired learning and memory (Sussel et al., 1999; Fragkouli et al., 2009; Malt et al., 2016; Magno et al., 2017)
	*Otx1*	Reduced cortex size; reduced cell population (Acampora et al., 1996; Pantò et al., 2004)	Medulloblastoma; spontaneous epilepsy and seizures (Boon et al., 2005; Zakrzewska et al., 2013)
	*Otx2*	Disrupted septum and cortex formation (Acampora et al., 1997)	Medulloblastoma (Boon et al., 2005; Zagozewski et al., 2020)
	*Pax6*	Disrupted cortex formation; thinned cortex; small eyes (Hill et al., 1991; Tyas et al., 2003; Quinn et al., 2007)	Autism; impaired audition; intellectual disability (Malandrini et al., 2001; Davis et al., 2008)
bHLH	*Ascl1*	Reduced *Dlx1/2* expression; impaired interneuron migration (Nieto et al., 2001; Bertrand et al., 2002)	Parkinson’s Disease (Ide et al., 2005)
	*Olig1*	Increased amount of GABAergic interneuron (Lu et al., 2000; Silbereis et al., 2014)	Down Syndrome (Haydar and Reeves, 2012)
	*Olig2*	Absence of OPCs (Furusho et al., 2006; Petryniak et al., 2007; Ono et al., 2008)	Down Syndrome; DMG (Lu et al., 2000; Filbin et al., 2018)
Forkhead	*Foxg1*	Reduced cortex size; impaired cortical cell proliferation (Xuan et al., 1995; Hanashima et al., 2002)	Autism; FoxG1 Syndrome; Rett Syndrome; schizophrenia; seizures; West’s Syndrome variants (Neul et al., 2010; Florian et al., 2011; Striano et al., 2011; Mariani et al., 2015; Won et al., 2016)

Aristaless Related Homeobox is expressed in various parts of the developing forebrain, such as the SVZ in developing GE and the VZ in the neocortex (Figure 1B; Miura et al., 1997; Colombo et al., 2004). *Arx* expression in the neocortex is limited to the proliferating neural progenitor cells, and is suppressed in cells radially migrating from the VZ (Friocourt et al., 2006), whereas in the GE *Arx* is continually expressed after neuronal differentiation and migration. Embryonic mice with homozygous *Arx* mutations have small brains with a thin neocortex and die upon birth, which may be related to defective tangential migration of cortical interneurons (Kitamura et al., 2002; Colombo et al., 2007; Friocourt et al., 2008). Targeted conditional deletion of *Arx* in the neocortex results in intermediate progenitor cell proliferation, with a reduced population of cortical neural progenitors. ARX also directly regulates cortical progenitor cell expansion through transcriptional regulation of CDKN1C, a cell cycle progression inhibitor in cortical VZ and SVZ (Colasante et al., 2015). The expression pattern of *Arx* also reveals its contribution to establishing the dorsoventral identity of the developing brain, where *Arx* suppresses ventralization in the dorsal forebrain by repressing *Olig2* expression. *Olig2* is a ventral specific gene and its expression is induced through Sonic Hedgehog (SHH) signaling. The expression of SHH downstream targets, *Gli1* and *Ptch3*, are increased in *Arx* cKO mice dorsal telencephalon. Thus ARX represses these SHH downstream signals, and in turn represses *Olig2* expression (Lim et al., 2019). Both inactivation, through shRNA, and overexpression of *Arx* impact GABAergic interneuron tangential migration to the neocortex from the MGE (Colombo et al., 2007). Furthermore, *Arx* is a direct regulatory target of DLX2, another homeobox transcription factor that regulates tangential migration, where overexpression of *Dlx2* increases *Arx* levels and reduction of *Dlx2* expression reduces *Arx* in the GE (Cobos et al., 2005a). By gain- and loss-of-function analysis, *Arx* was demonstrated to mediate the tangential interneuronal migration driven by DLX2, but not GABAergic neuron specification (Colasante et al., 2008). Conditional deletion of *Arx* in the ventral telencephalon further supports a role for *Arx* in tangential migration resulting in an overall reduction in the number of mature interneurons (Marsh et al., 2016). Additionally, ARX has been shown to transcriptionally regulate genes important for migration, such as *Cxcr4, Cxcr7*, *Ebf3*, and *Lhx7* (Fulp et al., 2008; Colasante et al., 2009; Quille et al., 2011).

Aristaless Related Homeobox mutations can lead to severe neurological diseases, including X-linked intellectual disability, epilepsy, as well as structural brain malformations (Table 3; Friocourt and Parnavelas, 2010), and these mutations have been studied extensively using mouse models (Kitamura et al., 2002, 2009; Marsh et al., 2009; Price et al., 2009). The phenotypes related to *ARX* mutations can be grouped based on whether there is a corresponding malformation. Disorders in the malformation group include X-linked lissencephaly associated with abnormal genitalia (Kitamura et al., 2002) and Proud syndrome (Kato et al., 2004), whereas the non-malformation group includes epilepsy, non-syndromic X-linked intellectual disability, and X-linked Infantile Spasms Syndrome (Bienvenu et al., 2002; Kitamura et al., 2009; Price et al., 2009) and different epilepsy syndromes such as West syndrome (Strømme et al., 2002; Kato et al., 2003). Many mutations in *ARX* have been found in the first two polyA tracts, where the polyA tracts are expanded by insertion of either additional alanine or other residues (Kitamura et al., 2009). A common mutation consists of an in-frame 24bp duplication (Szczaluba et al., 2006), whilst longer mutations, 27bp, and 33bp have also be reported (Demos et al., 2009; Reish et al., 2009). The longest known mutation exhibits the addition of eleven alanine residues, resulting in Ohtahara syndrome (Kato et al., 2007). Other intellectual disability, seizures related disorders have also been observed (Turner et al., 2002). In summary, these *ARX* mutations disrupt DNA and protein binding ability, perturbing the transcriptional activity of ARX, thereby affecting cortical development (Nasrallah et al., 2012; Siehr et al., 2020).

### Cut-Like Homeobox Genes

The Cut-like homeobox genes encode a transcription factor family [*Cux homeobox 1/2* (*Cux1/*2)], previously called *CCAAT-displacement protein (CDP) or Cut-like homeobox 1/2 (Cut1/2)*, that are the mammalian homologs of the *Drosophila* gene *cut* locus (*ct*) (Blochlinger et al., 1988). *Ct* is responsible for controlling the fate of neuronal progenitor cells in the peripheral nervous system and external sensory organs in *Drosophila* (Bodmer et al., 1987; Blochlinger et al., 1988) and plays a crucial role in dendritic arborization of specific sensory neurons (Grueber et al., 2003). *CUX1* is located on human chromosome 7q22 and is frequently rearranged in cancers (Scherer et al., 1993), while *CUX2* is on chromosome band 12q24.11-q24.12 (Craddock et al., 1993). CUX transcription factors contain up to four DNA binding regions, comprised of one HD, including a histidine residue at the 9^th^ amino acid of the third helix (Blochlinger et al., 1988), and one, two, or three highly homologous Cut repeats of approximately 70 amino acids (CR1, CR2, CR3) (Figure 2A; Nepveu, 2001). However, individual Cut repeats are unable to bind to DNA on their own but interact with other Cut repeats or with the Cut HD to bind DNA (Moon et al., 2000). CR1/CR2 mediate transient binding to DNA (Moon et al., 2000) and the CR3 repeat and the HD have been reported to form bipartite high affinity DNA binding interactions (Harada et al., 1994, 1995). *Cux1* and *Cux2* splice variants encode for protein isoforms with different combinations of DNA binding domains (Weiss and Nieto, 2019). Proteolytic cleavage of the full length p200 CUX1 protein generates a p110 protein which contains CR2, CR3 and the HD (Goulet et al., 2004). While the full-length p200 protein acts as a transcriptional repressor, p110 can act as repressor or activator depending on the type of promoter it interacts with (Yoon and Chikaraishi, 1994; Truscott et al., 2004, 2007; Harada et al., 2007). CUX proteins can act as transcriptional repressors either indirectly by competing with transcriptional activators for binding to target sites, or actively suppressing transcription *via* a mechanism that involves recruiting histone deacetylases through the Ala, Pro-enriched carboxyl domain (Cowell and Hurst, 1994; Mailly et al., 1996; Nepveu, 2001). CUX transcriptional activity is regulated by post-translational modifications at the Cut repeats which include acetylation, proteolysis (Sansregret et al., 2010), and phosphorylation by PKC (Coqueret et al., 1996), CKII (Coqueret et al., 1998), cAMP-dependent protein kinase (Michl and Downward, 2006), and cyclin A/Cdk1 (Santaguida et al., 2001), which repress transcriptional activity.

*Cux1* expression is detected widely in embryonic and adult tissues (Nieto et al., 2004), while *Cux2* is more specifically expressed in the nervous system (Quaggin et al., 1996) as well as the limb buds and urogenital system (Iulianella et al., 2003). *Cux1 and Cux2* are expressed early during brain development in neural progenitor cells in the ventral and dorsal telencephalon, as early as E14 for *Cux1* and E10.5 for *Cux2*, specifically *Cux1* is expressed in the VZ and SVZ of whole GE (Nieto et al., 2004; Zimmer et al., 2004; Figure 1). In contrast, *Cux2* is solely expressed in the SVZ of the MGE, and is enriched in tangentially migrating cortical interneurons (Nieto et al., 2004; Zimmer et al., 2004). Indeed, *Cux2* is mostly expressed in SVZ/IZ early during development while it is later expressed across most of the cortex (Zimmer et al., 2004). Furthermore, *Cux2* expression distinguishes two cortical neuronal subpopulations with different origins, migration models, and phenotypic characteristics: a population of tangentially migrating GABAergic cortical interneurons and another DLX-negative neuronal population produced in the pallium, which migrates radially, divides in the SVZ and accumulates in the IZ (Zimmer et al., 2004).

In addition to controlling neural specification and differentiation in upper cortical layers, CUX proteins can act as repressors for developmental processes such as dendritic arborization (Grueber et al., 2003; Cubelos et al., 2010; Li et al., 2010). Overexpression of *Cux1*, but not *Cux2*, results in decreased dendritic arborization in cultured cortical pyramidal neurons, whereas dendritic complexity increases upon reduction of *Cux1* (Li et al., 2010). A mechanism whereby *Cux1* transcriptionally represses dendritic arborization is through suppression of the cyclin-dependent kinase inhibitor p27^Kip7^ and further plays a role in proliferating cells by repressing the p21 cyclin kinase inhibitor (Coqueret et al., 1998).

*Cux2* is regulated by PAX6 and contributes to determining the upper layers (II-IV) of the cortex (Zimmer et al., 2004). Deletion of either *Cux1* or *Cux2* in mice does not alter overall cortical and brain organization (Cubelos et al., 2008a), whereas most *Cux1* and *Cux2* double homozygous mutants die prior to birth (Cubelos et al., 2008b). Although, the few pups that survive P0 do not display defects in neuronal migration or in layer specific protein expression (Cubelos et al., 2008b), *Cux1/Cux2* double knockout (DKO) mice display abnormal dendrites and synapses indicating a critical role for *Cux* genes in dendritogenesis (Cubelos et al., 2010). The formation of cortical interneurons in *Cux* single and double mutants is impaired while loss of Reelin expression is only observed in upper cortical layers II-IV in double mutants (Cubelos et al., 2008b).

*Cux2* deficient mice display increased brain volume, cell density and thickness of the upper cortical layers (II-IV), caused by an increase in the number of neuronal progenitor cells (Cubelos et al., 2008a). CUX1 target genes include *Nfib*, *Fezf2*, *Pou6f2* and *Sox5* which are all transcriptional regulators highly expressed in lower layers of the cortex (Gray et al., 2017). In addition to regulating upper cortical layer formation, *Cux2* has also been shown to control cell cycle exit (Cubelos et al., 2008a). Therefore, *Cux1* and *Cux*2 regulate neuronal proliferation of intermediate neuron precursors in SVZ, as well as the proliferation rate of neuronal precursor cells fated to form pyramidal cortical neurons in the upper layers of the cortex (Cubelos et al., 2008a,b) and in the spinal cord (Iulianella et al., 2008).

Mutations in *CUX1* have been associated with global developmental delay with or without impaired intellectual development (GDI) (Platzer et al., 2018) while *CUX2* is associated with intellectual disorders, seizures, autism spectrum disorder and bipolar affective disorder (Glaser et al., 2005; Barington et al., 2018). *CUX1* has also been shown to undergo inactivating mutations and loss of heterozygosity (LOH) in a number of human cancers (Ramdzan and Nepveu, 2014; Wong et al., 2014). Loss of *CUX1* activates the phosphoinositide-3-kinase (PI3K) signaling pathway as a result of transcriptional downregulation of the PI3K inhibitor, PIK3Ip1 (Wong et al., 2014). This mutation in *CUX1* results in increased tumor growth and increased susceptibility to PI3K-Akt inhibition (Wong et al., 2014). CUX1 has also been implicated in the regulation of proteosome-mediated degradation of the Src tyrosine kinase resulting in altered tumor cell migration and invasion (Aleksic et al., 2007).

### Distalless Genes

*Distalless* (*dll)* was discovered in *Drosophila* for its essential role in limb development (Cohen et al., 1989). *Dlx* genes are the vertebrate orthologs of *dll*; six members of this gene family can be found in humans and mice, occurring as bigenic clusters (*Dlx1/2, Dlx3/4*, *and Dlx5/6*); however, only *Dlx1*, *Dlx2*, *Dlx5*, and *Dlx6* are expressed in the forebrain (Figure 1B). *Dlx1/2* and *Dlx5/6* are located on mouse chromosomes 2 and 6, and on human chromosomes 2q31.1 and 7q21.3, respectively (Stock et al., 1996). These bigenic clusters are organized from tail-to-tail, with highly conserved intergenic enhancers located between the two genes. *Dlx1/2* and *Dlx5/6* each contain two intergenic enhancers: i12a and i12b for *Dlx1/2*, and i56a and i56b for *Dlx5/6* (Ghanem et al., 2003; Ruest et al., 2003). These *cis*-regulatory elements, although dissimilar in sequence, have overlapping activity and are essential for the expression of these genes (Fazel Darbandi et al., 2016). *Dlx5/6* expression is regulated by *Dlx1/2*, where the absence of *Dlx1/2* reduces *Dlx5/6* expression through the intergenic enhancer, revealed using gene reporter systems (Zerucha et al., 2000; Zhou et al., 2004). Likewise, removing the intergenic enhancers with a targeted mutation attenuates *Dlx5/6* expression in the forebrain, suggesting these intergenic enhancers are necessary for *Dlx* expression (Robledo et al., 2002; Ghanem et al., 2003). *Dlx* transcription factors are expressed in the developing GE and are essential for forebrain development (Pleasure et al., 2000). From embryonic day 9.5 (E9.5), expression is induced in the order of *Dlx2*, *Dlx1*, *Dlx5*, and *Dlx6* (Eisenstat et al., 1999). In mice, *Dlx1/2* are expressed in the VZ in the GE, and are clearly separated at the pallio-subpallial boundary (Figure 1B). *Dlx5/6* are expressed in the MZ of the ventral telencephalon, and additionally, *Dlx1, Dlx2, and Dlx5* are expressed in the SVZ in an overlapping manner, coinciding with regions where GABAergic interneurons are produced (Liu et al., 1997; Acampora et al., 1999; Depew et al., 1999; Robledo et al., 2002; Cobos et al., 2005b; Weinschutz Mendes et al., 2020).

*Dlx1* and *Dlx2* single gene homozygous knockout (KO) mice die prematurely at postnatal day 0 (P0) with minor abnormalities in GABAergic neuron formation, demonstrating DLX1 and DLX2 are somewhat functionally redundant (Qiu et al., 1997). Cortical neurons are reduced in postnatal *Dlx1* KO mice which can lead to seizures (Cobos et al., 2005b). *Dlx1/2* and *Dlx5/6* double homozygous mutants also die at P0 with a more significant forebrain defect compared to the single KO mice. Tangential interneuron migration from the MGE to the neocortex is blocked in *Dlx1/2* double homozygous mutants both in mice and zebrafish, hindering GABAergic interneuron development (Anderson et al., 1997a,b; MacDonald et al., 2013). *Dlx1/2* double homozygous mutants also have reduced *Dlx5/6* expression, which results in altered progenitor cell fate in the dorsal and ventral telencephalon (Pla et al., 2017). *Dlx5/6* double homozygous mutant mice also exhibit tangential migration defects, with poor specification of parvalbumin GABAergic interneuron subtypes (Wang et al., 2010). Therefore, *Dlx* genes are essential for the differentiation of GABAergic neurons and their subsequent tangential migration (Anderson et al., 1997a,b; Marin et al., 2000).

Distalless genes transcription factors promote interneuron production by regulating transcription of various downstream targets in the ventral telencephalon, binding to the core HD DNA binding motif ATTA/TAAT (Zhou et al., 2004; Table 2). A recent report has found that DLX2 binds preferentially to transcription factors to mediate both its’ repression and activation functions (Lindtner et al., 2019). GABA is synthesized by glutamic acid decarboxylases 1 and 2 (GAD1; GAD2) which are co-expressed with *Dlx1/2* in the VZ and SVZ of the GE in mouse, zebrafish, and humans (Erlander et al., 1991; Liu et al., 1997; Martin et al., 2000; MacDonald et al., 2010; Al-Jaberi et al., 2015). *Gad1* and *Gad2* expression is dependent on the DLX factors, where DLX1/2 bind directly to the promoters of *Gad1/2 in vivo* and induce *Gad1/2* expression; *Gad* expression is reduced in *Dlx1/2* double homozygous mutant mice (Stühmer et al., 2002; MacDonald et al., 2010; Li et al., 2012a; Le et al., 2017). However, *Gad* expression is not completely ablated in these mutants, which could be due to the compensatory function of residually expressed DLX5/6. Additionally, DLX proteins promote the differentiation of GABAergic and cholinergic interneuron subtypes through regulation of *Lhx6* and *Lhx8*, where both *Lhx* genes have reduced expression in *Dlx1/2* double homozygous mutants (Petryniak et al., 2007; Long et al., 2009). DLX2 also downregulates *Olig2* expression to repress oligodendrocyte development in early neurogenesis, and hence may control the balance between oligodendrocyte and neuron production (Petryniak et al., 2007; Jiang et al., 2020). ASCL1, in turn, represses *Dlx2* expression in later developmental stages, to allow the expression of *Olig2* and promote oligodendrocyte production (Petryniak et al., 2007; Poitras et al., 2007). The repression of *Olig2* by DLX2 also represses the promotion of the progenitor cell states, and likewise DLX2 downregulates several other transcription factors with similar functions, such as *Gsx2*, *Otx2*, and *Pax6* (Yun et al., 2001; Hoch et al., 2015; Lindtner et al., 2019). SMAD transcription factors, which are part of the transforming growth factor-β (TGF-β) signaling pathway, interact with DLX2 in binding to the promoter regions of DLX2 target genes in the telencephalon. Although expression of TGF-β signaling components is unaffected in the *Dlx1/2* double mutants, the interaction between DLX2 and SMAD factors indicate TGF-β could play a role in GABAergic interneuron differentiation (Shi and Massagué, 2003; Maira et al., 2010).

In addition to cell differentiation, DLX transcription factors also regulate interneuron tangential migration. DLX1/2 regulates this process by repressing terminal differentiation of interneurons (Cobos et al., 2007). Interneurons develop axons and dendrites post migration, promoted by proteins that regulate cytoskeleton and cell motility such as MAP2 and PAK3 (Anderson et al., 1997b; Bokoch, 2003; Dehmelt and Halpain, 2004). In *Dlx1/2* DKO mice, interneurons have significantly reduced migration, increased neurite length, and upregulated expression of genes which are normally expressed post-migration. Hence, DLX1/2 represses these genes to enable tangential migration of interneurons to the cortex (Cobos et al., 2007). *Nrp2*, encoding for a Semaphorin-3A and 3F receptor, is also repressed by DLX1/2, as evident in the marked increase of NRP2 expression in the forebrains of *Dlx1/2* DKO mice (Le et al., 2017). In *Dlx5/6* double homozygous mutant mice, a receptor for tangential migration *Cxcr4* is downregulated in the SVZ, which likely contributes to the impaired tangential migration observed in these mutants (Wang et al., 2010, 2011b).

Although DLX transcription factors have not been directly linked to any neurological diseases, many associations have been made between DLX mutations and neurodevelopmental defects (Table 3). Epilepsy and Rett syndrome had been linked to *Dlx* mutations in mouse models. Furthermore, *DLX1/2* and *DLX5/6* are found on chromosomes 2q and 7q, which are autism susceptibility loci (Cobos et al., 2005b; Hamilton et al., 2005; Horike et al., 2005). By site-directed mutagenesis of the *Dlx1/2* intergenic enhancer regions, transgenic mice with autism-like phenotypes were generated, showing the possible role of disrupted *Dlx1/2* in autism development (Poitras et al., 2007). Several neurodevelopmental disorders have been related to *Dlx* genes due to the importance of this gene family in regulating GABAergic interneuron production and migration (Kato and Dobyns, 2004; Verret et al., 2012). A DLX2 direct target *Grin2b* is linked to schizophrenia, epilepsy, intellectual disability, and autism, which provides evidence that DLX2 may contribute to neural diseases (Endele et al., 2010; Pan et al., 2019). DLX2 regulation of transcription factors such as *Arx* and *Olig2* also support that DLX factors may potentially contribute to neurological disease (Lindtner et al., 2019).

### Empty Spiracle Genes

*Empty spiracles homeobox* (*Emx)* genes are the mammalian homologues of the *Drosophila* gene *empty spiracle* (*ems*), which is responsible for head structure development (Younossi-Hartenstein et al., 1997). *Emx1* and *Emx2* are homeobox genes important for dorsal patterning in the forebrain. From mouse studies, *Emx2* is shown to be expressed earlier, from E8.5, whereas *Emx1* is expressed from E9.5 (Simeone et al., 1992; Gulisano et al., 1996; Medina and Abellán, 2009). The expression of *Emx2* is regulated by two sets of enhancers, one at the 5′ region and the other at the 3′ region (Theil et al., 2002; Suda et al., 2010; García-Moreno and Molnár, 2015). *Emx2* expression is directly promoted by DMRT5 and downregulated by the *Emx2* antisense transcript *Emx2OS* (Spigoni et al., 2010; Saulnier et al., 2013). Both *Emx* genes are expressed in the dorsal telencephalon, with the highest level of expression rostrolaterally, and decreased expression in a gradient caudomedially (Mallamaci et al., 1998). *Emx1* expression is nested within *Emx2* expression, and only *Emx2* is expressed in the caudomedial part of dorsal telencephalon (Simeone et al., 1992; Yoshida et al., 1997). While *Emx2* expression is restricted to progenitor cells, *Emx1* is expressed in both progenitor and differentiated cells (Gulisano et al., 1996).

Both *Emx1* and *Emx2* are necessary for the development of the archipallium in the dorsal telencephalon, and especially the development of the hippocampus and cortex in later stages (Simeone et al., 1993; Pellegrini et al., 1996; Yoshida et al., 1997; Hamasaki et al., 2004). *Emx1* and *Emx2* double homozygous mutants do not develop the dorsomedial telencephalon, whilst this phenotype is not observed in *Emx1* or *Emx2* single homozygous mutants (Bishop et al., 2003; Shinozaki et al., 2004). The impaired development of the neocortex could also be due to impaired tangential migration, as interneurons in *Emx1*/*Emx2* double mutant cannot migrate out of the GE into the cortex (Shinozaki et al., 2002). Homozygous *Emx1* mutants do not develop significant defects in the embryonic neocortex (Yoshida et al., 1997; Bishop et al., 2002). However, postnatal studies have shown that *Emx1* could play a role in cortical patterning, as rostral areas were expanded and caudal areas were reduced in the *Emx1* null mice (Stocker and O’Leary, 2016). *Emx1* homozygous mutants also lack development of the corpus callosum, and heterozygous *Emx1* mutants exhibit partial penetrance (Qiu et al., 1996). However, *Emx2* homozygous mutants have reduced neocortex size by E11.5, with defective dorsal telencephalon development, including aberrant hippocampus formation and impaired radial migration of neurons (Pellegrini et al., 1996; Yoshida et al., 1997; Mallamaci et al., 2000). In these mutants, there is ventralization of the dorsal telencephalon with reduced dorsal gene marker expression (*Ngn1*, *Ngn2*, and *Emx1*) and increased ventral marker gene expression (*Gsx2*, *Ascl1*, and *Dlx1/2*). *Emx2*/*Pax6* double homozygous mutants demonstrate a stronger phenotype with a lack of dorsal identity, showing these two homeobox factors function cooperatively to specify dorsal telencephalic identity (Muzio et al., 2002a,b). Furthermore, reciprocal inhibition is observed between *Emx2* and *Pax6*, where the cKO of one factor results in the upregulation of the other (Muzio et al., 2002b).

EMX1 and EMX2 regulate a number of factors required to specify dorsal telencephalic identity (Table 2). An important aspect of EMX2 function is its’ regulation of the formation of the PSB, along with PAX6 and GSX2 (Yun et al., 2001; Muzio et al., 2002a,b). EMX2 cooperates with DMRT5 and DMRT3 to repress *Gsx2* expression, with all three proteins binding directly to the ventral telencephalon specific *Gsx2* enhancer, thereby contributing to the development of the PSB (Desmaris et al., 2018). A mutual repressive relationship between EMX2 and FGF8 also promotes the differentiation of neural progenitor identity, where EMX2 downregulates FGF8 to promote differentiation, whilst FGF8 represses EMX2 to promote anterior-posterior patterning (Fukuchi-Shimogori and Grove, 2001, 2003; Cholfin and Rubenstein, 2008). In addition, EMX2 represses *Sox2* by inhibiting positive regulators from binding to *Sox2* enhancers. *Sox2* cKO mutants have a defective hippocampal phenotype, rescued when one *Emx2* allele is lost (Mariani et al., 2012). This demonstrates that EMX2 regulates hippocampal development, consistent with the observed *Emx2* homozygous phenotype (Pellegrini et al., 1996). Wnt signaling promotes *Emx2* expression through activation of an *Emx2* telencephalic enhancer, through the Wnt downstream factor GLI3 (Theil et al., 1999, 2002; Muzio et al., 2005). Additionally, EMX2 restricts *Wnt-1* expression in the forebrain, which is essential for maintaining normal neuronal radial migration (Iler et al., 1995; Ligon et al., 2003). EMX1 regulates *Nrp1*, an axonal guidance receptor that regulates cortical connectivity (Wright et al., 2007; Lim et al., 2015). Furthermore, EMX2 regulates Teneurin-1, a transmembrane protein that also functions in axonal guidance, through binding to an alternative promoter (Table 3) and promoting the transcription of an alternative transcript (Drabikowski et al., 2005; Li et al., 2006; Beckmann et al., 2011).

While EMX2 functions in promoting cell differentiation in the developing brain, it is considered as a possible tumor suppressor in different cancers, such as sarcoma, colorectal cancer, gastric tumors, and glioblastoma (Table 3; Li et al., 2012b; Aykut et al., 2017; Jimenez-García et al., 2021a,b). In many tumors, *EMX2* expression is downregulated due to methylation of the *EMX2* promoter (Okamoto et al., 2010; Qiu et al., 2013). *EMX2* over-expression blocks cell proliferation through inhibiting the canonical Wnt pathway, and also leads to cell cycle arrest with increased cell death of glioblastoma cells (Falcone et al., 2016; Monnier et al., 2018; Jimenez-García et al., 2021a).

### Genomic Screened Homeobox Genes

*Genomic screened homeobox* (*Gsx*, formerly *Gsh*) genes encode a family of transcription factors important for patterning of the ventral telencephalon. *Gsx* genes are the mammalian orthologues of the *Drosophila intermediate neuroblasts defective* (*ind*) genes; mutation in *Drosophila* induces a loss of intermediate neuroblasts (Weiss et al., 1998). GSX proteins bind to DNA *via* the homeobox domain (Figure 2), and GSX2 activity may depend on its dimerization state, where homodimers promote gene activation, and monomers enhance gene repression (Salomone et al., 2021).

*Gsx1* and *Gsx2* are widely expressed in the neural progenitors found in the VZ of the LGE (Toresson et al., 2000). *Gsx2* is mostly expressed in the dLGE with lower levels in the vLGE with complementary patterns for *Gsx1*, localizing to the vLGE and MGE (Toresson and Campbell, 2001; Yun et al., 2001). *Gsx1* and *Gsx2* are partially functionally redundant, due to similarities in their consensus DNA binding sites (Hsieh-Li et al., 1995; Valerius et al., 1995; Toresson and Campbell, 2001; Pei et al., 2011). *Gsx2* and *Gsx1/Gsx2* DKO mice have a reduced LGE size, with decreased number of olfactory bulb neurons as well as GABAergic interneurons (Yun et al., 2003). Dorsal markers including *Pax6* and *Ngn2* also expanded ventrally in *Gsx2* homozygous mutants (Szucsik et al., 1997; Corbin et al., 2000; Toresson et al., 2000; Yun et al., 2001). The expression patterns of *Pax6* and *Gsx2* are complementary, separated by the PSB, and these genes function cooperatively to define the dorsoventral identity of the developing forebrain (Figure 1B; Yun et al., 2001; Carney et al., 2009). *Gsx2* expression is repressed by a number of genes in the dorsal telencephalon, including *Pax6, Emx2, Dmrt3* and *Dmrt5* (Muzio et al., 2002a,b; Desmaris et al., 2018).

*Gsx2* regulates specification of neurons, oligodendrocytes and glial cells in the LGE (Kessaris et al., 2006; Fogarty et al., 2007; Chapman et al., 2018). Neurogenesis and oligodendrogenesis take place in the dLGE and vLGE, respectively, and are tightly controlled by *Gsx2* in a time-dependent manner. Conditional knockout (cKO) of *Gsx2* upregulates the oligodendrocyte precursor cell (OPC) marker *Pdgfrα*, and promotes premature oligodendrocyte differentiation (Corbin et al., 2003; Chapman et al., 2013, 2018). *Ascl1*, a bHLH transcription factor crucial for neurogenesis, has reduced expression levels in *Gsx2* homozygous mutant mice (Chapman et al., 2013). Studies have shown that *Gsx2* upregulates *Ascl1* in earlier embryo stages, promoting neuronal differentiation in early embryonic stages (Méndez-Gómez and Vicario-Abejón, 2012; Chapman et al., 2013; Wang et al., 2013). Since *Ascl1* promotes the NPCs to differentiate into interneurons, GSX2 inhibits ASCL1 activity to regulate the balance between progenitor cell proliferation and differentiation (Roychoudhury et al., 2020). Whilst *GSX2* upregulates *Ascl1*, it inhibits the homo- and heterodimer formation of ASCL1 essential for its DNA binding ability (Johnson et al., 1992; Nakada et al., 2004; Roychoudhury et al., 2020). In earlier embryonic stages (E9-11), *Gsx2* promotes striatal projection neuron specification from the vLGE, and in later embryonic stages (E12.5–E15) olfactory bulb interneurons are specified in the dLGE (Waclaw et al., 2010).

Overexpression of *Gsx2* from E13.5 promotes the specification of dLGE over vLGE, and subsequently favors neurogenesis over oligodendrogenesis (Waclaw et al., 2010; Pei et al., 2011; Chapman et al., 2013). GSX1 in *Ascl1* expressing progenitor cells represses *Gsx2* and promotes the maturation of NPCs by transitioning these cells from the VZ to the SVZ and induces differentiation (Pei et al., 2011). *Gsx1/Gsx2* DKO mice have expanded OPCs comparable to *Gsx2* homozygous mutants (Chapman et al., 2018). However, the reduced proliferation of OPCs in the *Gsx1/2* DKO compared to *Gsx2* homozygous mutants suggests GSX1 functions in promoting OPC proliferation in the ventral telencephalon (Chapman et al., 2018). Hence, *Gsx2* regulates neurogenesis through repressing *Gsx1*, and blocks oligodendrogenesis in early embryonic stages. Furthermore, downregulation of *Gsx2* in late embryonic stages is essential for oligodendrogenesis to proceed, which could be a result of negative autoregulation (Salomone et al., 2021).

Along with promoting *Ascl1* expression, GSX2 regulates neural differentiation *via* increasing *Dlx1* and *Dlx2* expression in the LGE (Table 2; Corbin et al., 2000; Toresson et al., 2000; Wang et al., 2013). *Dlx1/Dlx2* are part of the gene regulatory network downstream of *Ascl1* and in turn negatively regulate *Gsx1* and *Gsx2* expression (Yun et al., 2002; Long et al., 2009; Wang et al., 2013). The activation of *Gsx1* and *Gsx2* regulates the patterning of LGE, and later silencing of these two genes by *Dlx1/2* promotes subcortical neural differentiation (Anderson et al., 1997b; Cobos et al., 2005b). Furthermore, GSX2 also represses *Dbx1*, a homeobox transcription factor expressed in the hindbrain and spinal cord that regulates dorsoventral brain patterning and specification of Cajal-Retzius cells (Yun et al., 2001; Bielle et al., 2005; Winterbottom et al., 2010). DBX1 has also been suggested to repress *Gsx1* in the ventral telencephalon; however, further studies are necessary to validate this relationship (Poiana et al., 2020).

Congenital brain malformations may result from mutations in the *GSX2* gene (Table 3). Whole exome sequencing of patients with basal ganglia malformations reveals a homozygous missense mutation in *GSX2* HD that impair its transcriptional activity (De Mori et al., 2019). These patients have similar phenotypes to homozygous mutant mice models, with malformations or defective structures derived from the LGE and MGE (putamen, globus pallidus, caudate nucleus and olfactory bulb), as well as maldevelopment of the forebrain midbrain junction (De Mori et al., 2019). These anatomical defects are also associated with a range of neurological disorders, such as Parkinson’s and Huntington’s Diseases (Zuccoli et al., 2015; Table 2).

### Iroquois-Related Homeobox 3 Gene

*The iroquois-related homeobox 3* (*Irx3*) is a TALE HD containing transcription factor (Figure 2), orthologous to the *Iroquois*-complex genes in *Drosophila*, which are responsible for the development of sensory organ, body-wall and wing identity (Gómez-Skarmeta et al., 1996; Bürglin, 1997; Diez del Corral et al., 1999). *Irx* genes in vertebrates are organized into two clusters, *IrxA* and *IrxB*, each containing 3 genes from the family. The *IrxA* cluster consists of *Irx1, Irx2*, and *Irx4*, whereas *IrxB* contains *Irx3, Irx5*, and *Irx6*, located on mouse chromosome 8 and human chromosome 16 (Peters et al., 2000).

Iroquois-related homeobox 3 is important for thalamic patterning in the diencephalon (Robertshaw et al., 2013). *Irx3* is predominantly expressed in the midbrain, hindbrain, and spinal cord in early neurogenesis (E7.5–E9.5), and expression shifts rostrally to the diencephalon from E10.5 (Bosse et al., 1997). Notably, the expression patterns of *Irx3* and *Ascl1* during early neurogenesis are similar, which may suggest a regulatory relationship between the two transcription factors (Cohen et al., 2000). Similar to *Dlx1/2/5* and *Nkx2.1/2.2*, *Irx3* expression is posterior to the *zona limitans intrathalamica* (ZLI), a region in the diencephalon that releases SHH signaling molecules for the patterning of prethalamus and thalamus (Kitamura et al., 1997; Eisenstat et al., 1999; Robertshaw et al., 2013; Murcia-Ramón et al., 2020a). High levels of SHH signaling induces rostral thalamus, and subsequently the production of GABAergic interneurons, while a low level of SHH promotes caudal thalamus specification and glutamatergic interneurons production (Kiecker and Lumsden, 2005). Consistent with this, ectopic expression of *Irx3* promotes the expression of thalamus differentiation markers *Sox14* and Gbx2, both in the prethalamus and the dorsal telencephalon in response to SHH signaling (Kiecker and Lumsden, 2004; Robertshaw et al., 2013). However, such markers were not expressed upon *Irx3* ectopic expression in the ventral telencephalon, which may be due to SIX3 repression of *Irx3*, which restricts its activity to specify thalamus identity (Kobayashi et al., 2002; Robertshaw et al., 2013). In *Xenopus* models, knockdown of *Irx3* reduces midbrain size, and caudally shifts the forebrain-midbrain boundary, illustrating its function in ensuring the normal patterning of the diencephalon (Rodríguez-Seguel et al., 2009). A key co-regulator of thalamus patterning is PAX6, which is expressed anterior to the forebrain-midbrain boundary and specifies the caudal thalamus. The overlapping expression patterns of *Irx3* and Pax6 (see Figure 2A) mark the region of thalamus patterning, while caudal and rostral thalamus identity is determined by levels of SHH signaling (Robertshaw et al., 2013).

Iroquois-related homeobox 3 is considered to be a determinant for obesity, in relation to the fat mass and obesity associated (FTO) genes, due to the role of *Irx3* in neurogenesis at the paraventricular nucleus of the hypothalamus, developed from the anterior hypothalamus (Smemo et al., 2014). *Single-minded 1* (*Sim1*), a bHLH transcription factor in the hypothalamus represses *Irx3* expression, as *Sim1* KO mice exhibit ectopic expression of *Irx3* in the anterior hypothalamus (Caqueret et al., 2006; Son et al., 2021b). *Sim1* homozygous mutant mice are perinatal lethal, whereas *Sim1* heterozygous mutant mice exhibit neurodevelopmental defects and hyperphagia, as *Sim1* is important for neurogenesis in the hypothalamus (Michaud et al., 1998; Holder et al., 2004). The neurogenesis defects in these mutant mice are due to the ectopic expression of *Irx3* and *Irx5* in the anterior hypothalamus (Son et al., 2021a,b). In *Sim1/Irx3/Irx5* triple heterozygous KO mice, the neuronal population at the anterior hypothalamus is restored. Similarly, cKO of *Irx3* at the paraventricular nucleus of the hypothalamus partially rescues the neuronal disruption observed in *Sim1* heterozygous mutant mice, with no observable differences in body weight or hyperphagic phenotype (Son et al., 2021b).

### Lhx (LIM-HD Family) Genes

The *Lhx* transcription factors belong to the LIM-HD family of homeobox genes that have both a LIM zinc finger domains and a HD (Figure 2; Dawid et al., 1998; Bach, 2000). The LIM zinc finger domain is named after the first three genes discovered in the family, *Lin-11, Isl1* and *Mec-3*, and participates in protein-protein binding (Way and Chalfie, 1988; Freyd et al., 1990; Karlsson et al., 1990). Of the various members of the *Lhx* gene family found in both mouse and humans, *Lhx1, Lhx2, Lhx5, Lhx6*, and L*hx8 (i.e., L3/Lhx7)* are important for differentiation and migration of interneuron in the developing telencephalon (Alifragis et al., 2004; Abellán et al., 2010; Godbole et al., 2018). Mutant mice studies had provided insights into the importance of these *Lhx* genes for forebrain development (Wanaka et al., 1997).

*Lhx1* homozygous mutant mice have an increased number of PoA-derived interneurons and glia cells, suggesting *Lhx1* regulates the survival of these cells by regulating the balance between apoptosis and proliferation. Also the PoA-derived interneurons in *Lhx1* null mice migrate through the ventral telencephalon, compared to a more controlled migration in the wild-type mice through the developing neocortex (Symmank et al., 2019). *Lim5* is expressed in the forebrain of zebrafish and *Xenopus*, and *Lhx5*, the *Lhx1* paralog, is the murine ortholog. *Lhx5* is expressed predominantly in the hindbrain at E8, and the developing forebrain starting at E9.5. After E11.5, *Lhx5* is exclusively expressed in the ventral telencephalon, hypothalamus and diencephalon, which is complementary to *Dlx5* expression (Figure 1B; Sheng et al., 1997). Both *Lhx1/5* are expressed in the rostral area of the ZLI in the diencephalon, but only *Lhx1* is expressed in the caudal ZLI (Nakagawa and Leary, 2001). *Lhx5* homozygous mutant mice are defective in hippocampus development, where progenitor cells can proliferate but fail to exit the cell cycle to migrate or differentiate (Zhao et al., 1999). Cajal-Retzius neurons are responsible for the organization of the neocortex through the secretion of reelin (Soriano and Del Río, 2005). In mice, *Lhx5* regulates the development and migration of Cajal-Retzius cells, which could be critical to the malformation of the hippocampus in *Lhx5* null mutants (Abellán et al., 2010). *Lhx1* likewise is expressed in some Cajal-Retzius cells, but limited to the septal area, and lateral olfactory to caudomedial zones (Miquelajáuregui et al., 2010). Additionally, *Lhx5* can regulate forebrain development by suppressing Wnt signaling in zebrafish embryos, *via* promoting the expression of Wnt inhibitors *Sfrp1a* and *Sfrp5*, supported by the increase of Wnt signaling in zebrafish embryos lacking *Lhx5* expression (Peng and Westerfield, 2006). There is some evidence of *Lhx5* inhibiting Wnt5a in murine hypothalamus, promoting the growth of the mamillary body; however, more studies are required to confirm this regulatory effect and mechanism. Another possible target of *Lhx5* is *Lmo1* (LIM-only1), where *Lmo1* competes with *Lhx5* to bind with the *Lhx* binding partner LDB, thereby inhibiting *Lhx* function (Bach, 2000; Heide et al., 2015).

*Lhx2* is the mammalian ortholog of the *Drosophila apterous* gene, first described in 1913, as an essential gene for *Drosophila* wing development (Metz, 1914; Butterworth and King, 1965). *Lhx2* homozygous mutants have reduced forebrain volume, but expanded neocortex and PSB composing the entire forebrain (Porter et al., 1997; Bulchand et al., 2001; Monuki et al., 2001). *Lhx2* plays a role in suppressing hippocampus (hem) and PSB (antihem) development up to E9.5 and E10.5, respectively (Roy et al., 2014; Godbole et al., 2018). Suppression of hippocampal development is regulated by interactions between *Lhx2* and other transcription factors, namely *Foxg1* and *Pax6*. *Foxg1* has been shown to directly regulate *Lhx2* expression, where the loss of *Foxg1* also results in a loss of *Lhx2* at E9.5. cKO of *Lhx2* after E9.5 did not alter hippocampus development unless *Foxg1* was also knocked out (Godbole et al., 2018). *Pax6* is expressed in a lateral medial gradient in the neocortex, which is opposite to that of *Lhx2*. In *Pax6*/*Lhx2* DKO, the hippocampus expands more so in the forebrain compared to *Lhx2* null mice, suggesting *Pax6* also suppresses the formation of hippocampus (Godbole et al., 2017).

*Lhx6* and *Lhx8* are structurally related and have synergistic functions. *Lhx6* shares 75% homology with *Lhx8*, which is also known as *L3* or *Lhx7* (Matsumoto et al., 1996; Grigoriou et al., 1998). Both these genes are expressed overlappingly in the MGE but are not expressed in the LGE (Figure 1B). *Lhx6* is expressed predominantly in the SVZ and the MZ, whilst *Lhx8* is expressed in the MZ (Matsumoto et al., 1996). The expression of both these genes is regulated by *Nkx2.1*, another homeobox transcription factor that specifies ventral telencephalon development (Sandberg et al., 2016).

*Lhx6* has similar functions to *Lhx1*. *Lhx6* promotes expression of receptors that regulate cortical interneuron migration and transcription factors that control interneuron production, thereby regulating these events (Alifragis et al., 2004; Zhao et al., 2008; Neves et al., 2013). Tangential migration of GABAergic interneurons from the MGE into the neocortex are blocked in embryonic mice lacking *Lhx6;* normally these interneurons express *Lhx6* in wildtype mice (Lavdas et al., 1999; Alifragis et al., 2004; Liodis et al., 2007). Such migration defects prevent the formation of functional connections between these neurons and their post-synaptic targets. Since *Lhx6* has restricted expression in MGE progenitor cells, it does not regulate the migration of all cortical interneurons during development, especially at later stages where tangentially migrating neurons are born in the LGE (Marin et al., 2000; Nery et al., 2002). Production of GABAergic interneurons and their migration within the MGE are not affected in *Lhx6* mutants, but interneuron subtype specification is dependent on the expression of *Lhx6* (Neves et al., 2013). MGE-derived cortical interneurons are unable to differentiate into *sst*^+^ and *pva*^+^ subtypes, shown by a drastic reduction in the number of these neurons in *Lhx6* null mutants. *Lhx6* KOs had a greater effect on *sst*^+^ interneuron differentiation than *pva*^+^ interneuron differentiation, where *pva*^+^ interneuron differentiation was affected restrictively in the hippocampus (Liodis et al., 2007; Zhao et al., 2008; Yuan et al., 2018).

*Lhx8*, unlike *Lhx6*, is expressed in cholinergic neurons instead of GABAergic neurons (Lopes et al., 2012). *Lhx8* is essential for the differentiation and specification of cholinergic interneurons, shown by the reduction of cholinergic neurons in *Lhx8* homozygous mutant mice (Zhao et al., 2003; Fragkouli et al., 2005). Progenitor cells proliferate in *Lhx8* homozygous mutant mice; however, they are unable to differentiate into cholinergic interneurons or glutamatergic neurons (Manabe et al., 2007, 2008). Cholinergic neurons are derived from progenitor cells in the MGE, where LHX8 promotes the expression of *Isl1* upon cholinergic commitment, which in turn represses *Lhx6* expression (Zhao et al., 2003). *Lhx8* forms a hexamer with *Isl1* and promotes cholinergic neuron expression by binding to specific motifs in the cholinergic enhancer sequence (Park et al., 2012). The formation of hexamers is necessary for DNA binding and subsequently cholinergic gene expression, whilst LHX8 or ISL1 alone does not bind to cholinergic enhancer sequences and are unable to promote cholinergic interneuron differentiation (Cho et al., 2014). NPCs in the striatum differentiate into GABAergic interneurons instead of cholinergic neurons in *Lhx8* homozygous mutants. This is due to an upregulation of *Lhx6* as a result of a lack of *Isl1*, suggesting the necessity of *Lhx8* in cholinergic neuron specification (Manabe et al., 2005; Bachy and Rétaux, 2006). Additionally, *Lhx6* acts cooperatively with *Lhx8* to promote *shh* expression in the MGE, regulating the production of interneuron progenitors, as well as inhibiting *Nkx2.1* expression in cortical neurons (Flandin et al., 2011). The *Lhx6* and *Lhx8/Isl1* regulatory network is therefore essential for regulating the differentiation of GABAergic and cholinergic neurons in the ventral telencephalon.

The LIM-domain transcription factor family is functionally important for the specification, differentiation and migration of neurons in the developing forebrain, and mutations in these genes can result in genetic diseases (Table 3). *LHX2* mutations can result in pituitary hormone deficiency, although it is uncommon that a mutation in *LHX2* alone can cause pituitary deficiency and developmental ocular abnormalities (Prez et al., 2012). The importance of *Lhx6* on the differentiation of interneurons into *sst*^+^ and *pva*^+^ subtypes have a pathological link to schizophrenia (Volk et al., 2014; Donegan et al., 2020). There is reduced *LHX6* expression in schizophrenic subjects who also have reduced expression of *GAD1* (otherwise known as GAD67, a GABA synthesizing enzyme), *sst*, and *pva* expression. Reduction in *GAD1* does not downregulate *LHX6* and *vice versa*; hence, upstream factors likely contribute to the regulation of these genes (Volk et al., 2012). Moreover, a decrease in both GABAergic and cholinergic interneurons in the ventral telencephalon has been reported in Tourette Syndrome, suggesting *LHX6* and *LHX8* correlation with Tourette Syndrome due to their role in GABAergic and cholinergic interneuron specification in the striatum (Pagliaroli et al., 2020).

### Myeloid Ectopic Viral Integration Site 2 Gene

The *myeloid ectopic viral integration site* (*Meis)* gene family belongs to the TALE class of homeobox proteins, a homolog of the *Drosophila homothorax* gene, which is essential for directing the localization of *Pbx Drosophila* homologue *extradenticle* (Rieckhof et al., 1997). There are three mammalian MEIS transcription factors (*Meis1, Meis2, and Meis3*), and all contain a conserved homothorax domain (Figure 2A), which promotes the interaction between MEIS and pre-B cell leukemia homeobox proteins (PBX), a transcription factor known for its regulatory role in organogenesis (Nakamura et al., 1996; Chang et al., 1997; Golonzhka et al., 2015). MEIS proteins are characterized by a three residue loop insertion between helices 1 and 2 of the HD, an important feature for protein-protein interactions (Bürglin, 1997). Out of the three *Meis* genes, only *Meis1* and *Mei2* are expressed in the developing telencephalon (Figure 1B). *Meis2* in particular is an important player for striatal progenitors and neuron differentiation, as well as postnatal neuronal differentiation in the olfactory bulb (Toresson et al., 1999; Agoston et al., 2014).

Myeloid ectopic viral integration site 2 is expressed in the VZ of the entire telencephalon from E10.5, and is enriched in the LGE compared to the MGE from E12.5 to E18.5 (Figure 1B). From E14.5, MEIS2 is also expressed in the ventral thalamus and the anterior hypothalamus (Cecconi et al., 1997; Toresson et al., 1999, 2000). Additionally, the expression pattern of MEIS2 is similar in the human fetal forebrain, where MEIS2 is expressed in the proliferative zones (Larsen et al., 2010a). In the telencephalon, MEIS2 was initially considered as an LGE-specific marker due to its predominant expression in the LGE; however, MEIS2 is also widely expressed in the CGE progenitors (Toresson et al., 1999; Frazer et al., 2017). Postnatally, interneurons born and derived from the olfactory bulb express MEIS2, as it plays a crucial role, along with other transcription factors, in neuronal differentiation and specification in early postnatal stages (Allen et al., 2007; Agoston et al., 2014).

Myeloid ectopic viral integration site 2 forms complexes with various other transcription factors to cooperatively facilitate the expression of genes required for neurogenesis. As mentioned, MEIS2 interacts with PBX1 proteins and forms heteromeric complexes, which regulate the DNA binding ability of the two transcription factors (Liu et al., 2001; Longobardi et al., 2014). The MEIS2-PBX1 complex further recruits other transcription factors, such as the Kruppel-like factor 4 (*Klf4*) to modulate MEIS2 transcriptional activities (Bjerke et al., 2011). Other than PBX1, MEIS2 also functions synergistically with HOX and PAX homeobox factors, regulating the gene expression of other targets in the midbrain and hindbrain (Agoston et al., 2012). Mechanisms for the interactions between MEIS2 and other factors have been extensively reviewed; notably, MEIS2 recognizes and binds to a specific DNA motif TGACAG (Table 2; Chang et al., 1997; Longobardi et al., 2014; Schulte, 2014).

Myeloid ectopic viral integration site 2 controls gene expression and promotes neuronal migration and differentiation during forebrain development. There are three types of serotonin receptor 3a expressing (*Htr3a+*) GABAergic interneurons, which populate different regions of the brain. Type I *Htr3a*+ are enriched in transcription factors expressed in the LGE, including MEIS2, and these interneurons populate the deep cortical layers (von Engelhardt et al., 2011; Frazer et al., 2017). These interneurons originate from the PSB and migrate through to the cortex, contrasting with other types of *Htr3a*+ interneurons which are born from the CGE and populate the superficial cortical layers. Ectopic expression of *Meis2* in CGE born interneurons resulted in a shift of differentiated *Htr3a+* interneurons to the deep cortical layers, indicating that MEIS2 induces the migration of the LGE-derived interneurons (Frazer et al., 2017). Alternatively, MEIS2 can regulate expression of the *Dlx* family, by interacting with the intergenic enhancers in the *Dlx* bigenic clusters (Ghanem et al., 2003). MEIS2 binds to the I12b intergenic enhancer of *Dlx1/2* and the I56ii intergenic enhancer of *Dlx5/6*. MEIS2 can activate reporter gene transcription with a I56ii promoter sequence *in vitro* (Yang et al., 2000; Poitras et al., 2007; Ghanem et al., 2008). Subsequently, the removal of I56ii sequence reduced *Meis2 and Dlx5/6* expression, suggesting that there may be a positive feedback loop between MEIS2 and DLX5/6, further regulating interneuron migration (Fazel Darbandi et al., 2016). Furthermore, dopamine receptor expressing (D1/D2) MSNs are promoted by MEIS2 in the LGE, where deletion of *Meis2* blocked differentiation of neural progenitors and reduced the medium-spiny neuron population (Su et al., 2022). MEIS2 regulates specification of these striatal projection neurons through the promotion of *Zfp503* and *Six3* expression, while *Meis2* expression itself is regulated by DLX1/2 (Su et al., 2022). Likewise, in the prethalamus, DLX2 drives GABAergic interneuron determination through promoting *Meis2* expression, and SOX14 represses *Meis2* expression to maintain rostral thalamus identity (Sellers et al., 2014). In postnatal stages, interneurons continue to arise from the olfactory bulb SVZ generated neuroblasts where these differentiation events are dependent on the activity of MEIS2 and its interaction with PAX6 and DLX2 (Ming and Song, 2011; Agoston et al., 2014). Indeed, cKO of *Meis2* in the olfactory bulb blocks dopaminergic neuron differentiation, as MEIS2 promotes expression of *Dcx* and *Th*, both crucial genes for dopaminergic neuron subtype specification (Agoston et al., 2014; Kim et al., 2020).

### Nkx2.1/2.2 Genes

*Nkx2.1* and *Nkx2.2*, homeobox transcription factors of the vertebrate *Nkx* family, are important for the regulation of embryonic telencephalon and diencephalon patterning (Price et al., 1992; Sussel et al., 1999). *Nkx2.1* is the mammalian homolog of the *Drosophila scarecrow* (*scro*), and is also known as the thyroid transcription factor 1 and thyroid specific enhancer binding protein, since it also plays a role in thyroid, lung and pituitary development (Guazzi et al., 1990; Mizuno et al., 1991; Kimura et al., 1996; Maurel-Zaffran and Treisman, 2000). *Nkx2.2* is homologous to the *Drosophila ventral nervous system defective* (*vnd*) gene (Kim and Nirenberg, 1989; Price et al., 1992; Jimenez et al., 1995). *Nkx2.1* and *Nkx2.2* encode both a HD and a NK2 box domain (Figure 2). In embryonic forebrain, *Nkx2.1* is expressed in progenitor and post-mitotic cells in the MGE and PoA, and is essential for the patterning of these areas (Xu et al., 2005). *Nkx2.2* is localized to the MGE, the VZ of the thalamus and MZ of the diencephalon; however, *Nkx2.2* expression can vary in different mammalian species (Ericson et al., 1997; Flames et al., 2007; Vue et al., 2007; Bardet et al., 2010; Domínguez et al., 2015). The dorsoventral expression pattern of *Nkx2.1* (ventral) is complementary to that of *Pax6* (dorsal) (Figure 1B). Within the thalamus, *Nkx2.2* expression is induced by SHH signaling in the rostral thalamus along with *Ascl1*, resulting in the specification of GABAergic neurons that populate the thalamus; as a result, *Nkx2.2* is often co-expressed with SHH (Briscoe et al., 1999; Vue et al., 2007; Robertshaw et al., 2013).

*Nkx2.1* expressing progenitor cells give rise to GABAergic and cholinergic neurons, which populate the neocortex and striatum, respectively (Anderson et al., 2001; Magno et al., 2017). *Nkx2.1* expression in the GABAergic interneurons then diminishes after they tangentially migrate toward the neocortex, but is sustained in the cholinergic neurons (Marin et al., 2000). In the MGE, *Nkx2.1* silencing is necessary for interneurons to tangentially migrate. *Nkx2.1* silencing promotes the expression of *Nrp1* and *Nrp2*, which then initiates neural migration (Nóbrega-Pereira et al., 2008; Kanatani et al., 2015). *Nkx2.1* expressing neurons in the hypothalamus tangentially migrate into the diencephalon, and develop into GABAergic interneurons (Murcia-Ramón et al., 2020b). Additionally, NKX2.1 regulates astrocyte differentiation in the MGE and PoA from E14.5 to E16.5 in mice, and oligodendrocyte differentiation from E12.5 (Kessaris et al., 2006; Minocha et al., 2015, 2017; Orduz et al., 2019). Transcriptional activity is dependent on epigenetic states (Attanasio et al., 2014; Sandberg et al., 2016).

*Nkx2.1* homozygous mutant mice die at birth with lung, thyroid, pituitary and ventral telencephalon defects (Kimura et al., 1996; Takuma et al., 1998; Sussel et al., 1999). In these mutant mice, the MGE is respecified into LGE, and exhibits reduced numbers of GABAergic and cholinergic neurons (Sussel et al., 1999; Fragkouli et al., 2009). However, ∼50% of GABAergic interneurons remain, suggesting that NKX2.1 is not the sole factor required for GABAergic interneuron specification (Sussel et al., 1999). cKO of *Nkx2.1* at E10.5 and E12.5 results in altered identity of the MGE-derived interneurons subtypes. The MGE progenitor cells of these mutants were respecified into calretinin and vasointestinal peptide (VIP) expressing interneuron subtypes, resembling interneuron populations derived from the caudal GE (Xu et al., 2004; Butt et al., 2005), as opposed to *pva*^+^ or *sst*^+^ subtypes (Butt et al., 2008). GABAergic interneuron differentiation, especially *pva*^+^ and *sst*^+^ subtypes, is tightly regulated by *Lhx6* and *Lhx8* in the MGE, and both genes are downstream targets of NKX2.1 (Du et al., 2008; Flandin et al., 2011; Sandberg et al., 2016; Kim et al., 2021). *Lhx6* and *Lhx8* are activated by NKX2.1 expression in the SVZ through the recognition of epigenetic markers, and are essential for the specification of *pva*^+^ and *sst*^+^ interneuron subtypes (Du et al., 2008; Kim et al., 2021). Furthermore, NKX2.1 regulates MGE identity through repression of genes in the SHH, Wnt, and BMP signaling pathways required for cell differentiation and patterning. This repression is likely achieved by recruitment of Gro/TLE, a complex that reduces epigenetic-mediated repression, and induces activation (Patel et al., 2012; Sandberg et al., 2016). Conversely, SHH can induce the expression of *Nkx2.1* in the MGE to specify ventral identity (Ericson et al., 1995). To establish ventral identity in the telencephalon, NKX2.1 also represses *Pax6* expression in the GE, as the *Nkx2.1* cKO showed a dorsal to ventral expansion and ectopic expression of *Pax6* ventrally (Manoli and Driever, 2014). *Pax6*, a dorsal telencephalon specifying gene, in turn represses the expression of *Nkx2.1* in the neocortex. The existence of this mechanism of mutual repression is supported by the complementary expression patterns of these two transcription factors (Sussel et al., 1999; Stoykova et al., 2000).

As *NKX2.1* is essential for formation of various organs, mutations in this gene are linked to multiple phenotypes and diseases, including neurological disease, lung defects and thyroid dysfunction (Table 3; Thorwarth et al., 2014). *NKX2.1* may play a role in Hirschsprung disease, a disorder of the developing enteric nervous system, through its interaction with *SOX10* and *PAX3*. Sex-determining factor SRY is reported to displace SOX10’s interaction with NKX2.1 and PAX3, thereby promoting a Hirschsprung disease phenotype (Li et al., 2015). Furthermore, hereditary chorea, also known as brain-lung-thyroid disease, is linked to mutations in *NKX2.1* with symptoms such as impaired coordination or speech development (Krude et al., 2002; Monti et al., 2015). Subsequently, *NKX2.1* has also been related to the development of schizophrenia, as *Nkx2.1* regulates GABAergic and cholinergic neuron specification (Sussel et al., 1999; Fragkouli et al., 2009; Malt et al., 2016). The cholinergic specification function of *Nkx2.1* correlates with learning and memory, where the absence of *Nkx2.1* in the septal area results in cognitive impairments (Magno et al., 2017).

### Orthodenticle Homeobox Genes

*Orthodenticle homeobox* (*Otx*) is an ortholog of the *Drosophila orthodenticle* transcription factor, with *OTX1* located in the human chromosome region 2p13 and *OTX2* located in the human chromosome region 14q21-22 (Kastury et al., 1994). *Crx* is another member of the *Otx* family, but its expression is restricted to the retina, and all three *Otx* genes share a common OTX tail domain at the C-terminal (Figure 2A; Furukawa et al., 1997). *Otx1* plays an important role in cortical neurogenesis, and along with *Otx2*, both genes are important for forebrain patterning and specification, as well as retinal development (Larsen et al., 2010b). *Otx2* is essential during gastrulation for forebrain specification, and *Otx2* expression continues in both dorsal and ventral telencephalon, diencephalon, and mesencephalon (Acampora et al., 1995; Rhinn et al., 1998; Tian et al., 2002; Kurokawa et al., 2004; Sakurai et al., 2010). The midbrain/hindbrain boundary marks the caudal limit of *Otx2* expression. *Otx2* expression is repressed in the hindbrain and spinal cord (Frantz et al., 1994). This pattern is regulated by fibroblast-growth-factor (*Fgf*)-8 and *Gbx2*, another homeobox gene that is required for caudal brain patterning and formation (Garda et al., 2001). GBX2 recognizes a conserved enhancer sequence in *Otx2*, thereby downregulating *Otx2* in the hindbrain (Kurokawa et al., 2006; Inoue et al., 2012). *Otx1* and *Otx2* exhibit a similar expression pattern early in embryogenesis, and *Otx1* expression is nested within the *Otx2* expressing regions (Simeone et al., 1993). From E8.5, *Otx2* expression starts to diminish in the rostral forebrain. At E11.5, *Otx2* is expressed in the VZ in the GE, and promotes the ventral identity of the MGE. *Otx1* expression patterns change to become complementary to *Otx2*; it is predominantly expressed in the VZ of the dorsal telencephalon and is expressed at lower levels in the dLGE (Hoch et al., 2015; Huang et al., 2018).

Mice lacking *Otx1* survive to birth but develop spontaneous epilepsy and seizures (Acampora et al., 1996). cKO of *Otx1* in the developing neocortex reduces the size of the neocortex as well as the overall cellular population (Pantò et al., 2004). Deletion of *Otx1* reduced the generation of neurons by repressing neural differentiation from cortical NPCs, while NPC proliferation was promoted, subsequently increasing the population of neurons (Table 3). This suggests *Otx1* promotes cell cycle exit in cortical NPCs, thereby maintaining the balance between differentiation and proliferation (Huang et al., 2018).

Deletion of both *Otx1* and *Otx2* in mice results in a gastrulation defect and is embryonic lethal (Acampora et al., 1995). Heterozygous double mutants exhibit a range of phenotypes, including different degrees of craniofacial malformations, ocular defects, abnormalities in central nervous system, pituitary glands dysfunction, and developmental delay (Matsuo et al., 1995; Ang et al., 1996; Ragge et al., 2005; Tajima et al., 2009; Dateki et al., 2010; Mortensen et al., 2015). cKO of *Otx2* at different embryonic developmental timepoints and locations has shown a range of phenotypes indicating the essential role for *Otx2* in processes such as septum formation, specification of the neocortex, neurogenesis and early oligodendrogenesis and NPC fate (Acampora et al., 1997; Puelles and Rubenstein, 2003; Puelles et al., 2006; Silbereis et al., 2014; Hoch et al., 2015). The disruption of septum formation and cortex specification following cKO of *Otx2* after gastrulation suggests that *Otx2* could be regulating specification through FGF signaling (Acampora et al., 1997; Puelles and Rubenstein, 2003; Hoch et al., 2015). MGE interneuron markers such as *Dlx1*, *Arx*, and *Gbx* were downregulated in MGE-deleted *Otx2*, as well as the expression of oligodendrogenesis promoting genes *Olig1* and *Olig2* demonstrating a requirement for *Otx2* in neurogenesis and oligodendrogenesis (Silbereis et al., 2014; Hoch et al., 2015). Furthermore, *Lhx6* and *Lhx8* expression were reduced, suggesting *Otx2* plays a role in regulating cholinergic neurons. *Otx2* deletion in the thalamus resulted in a switch in NPC fate from glutamatergic neurons to GABAergic interneurons, demonstrating a requirement for *Otx2* in glutamatergic neuron specification (Puelles et al., 2006).

*OTX1* and *OTX2* are overexpressed in medulloblastoma (Boon et al., 2005; Zakrzewska et al., 2013), which is a malignant pediatric brain tumor located in the posterior fossa that is divided into four molecular groups based on genomic and transcriptomic alterations: Wnt, SHH, Group 3, and Group 4 (Rudin et al., 2009; Northcott et al., 2011; Taylor et al., 2012). *OTX2* is overexpressed in over 60% of medulloblastoma, usually in Groups 3 and 4 (Boon et al., 2005; Bunt et al., 2010). It has been postulated that the cellular context dependent nature of *OTX2* expression could attribute to its overexpression in some groups of medulloblastoma (Kaur et al., 2015). As *MYC*, another oncogene is also overexpressed in Group 3 medulloblastoma, OTX2 may promote tumorigenesis by cooperatively binding with MYC to target genes (Bunt et al., 2011). Furthermore, OTX2 promotes the proliferation of tumors in Groups 3 and 4 (Lu et al., 2017; Zagozewski et al., 2020). The overexpression pattern observed may be a result of autoregulation. Chromatin accessibility is altered in medulloblastoma, where histone modifications may allow for increased *OTX2* expression, and hence a positive feedback loop for *OTX2* (Wortham et al., 2014). Recent studies have also shown *OTX2* is potentially required for tumor proliferation in the SHH group, although not necessarily for tumor formation (El Nagar et al., 2018). In Group 3 medulloblastoma, OTX2 represses *PAX3* and PAX6. Overexpression of *PAX3* and *PAX6* is associated with increased patient survival (Zagozewski et al., 2020). Additionally, OTX1 and OTX2 have been shown to act as oncogenes, promoting tumorigenesis and proliferation for cancers such as hepatocellular carcinoma, breast cancer, and Hodgkin or non-Hodgkin lymphomas (Omodei et al., 2009; Terrinoni et al., 2011; Nagel et al., 2015; Li et al., 2016; Tu et al., 2020).

### Paired Box 6 Gene

The highly conserved *Pax6* transcription factor was first identified as a member of the *Paired box* (*Pax*) gene family based on its homology to the *Drosophila* gene *eyeless*. There are nine PAX transcription factors identified in mammals; all contain a paired domain and can be further categorized according to the presence or absence of additional domains, usually a HD. *Pax6* contains two DNA binding domains, a paired domain and a HD, as well as a proline-serine/threonine rich domain in the carboxyl-terminal (Figure 2A; Glaser et al., 1992; Duan et al., 2013). Hence, PAX6 binds to paired-HD and HD consensus DNA binding motifs (Sun et al., 2015). The *Pax6* homologue *eyeless* was first described in *Drosophila* as a gene essential for segmentation and eye development (Walther et al., 1991; Gehring, 1996), and in mammals it is important for the development of the CNS, eyes, pancreas, and pituitary gland (Dohrmann et al., 2000; Jones et al., 2002). In mice, *Pax6* expression begins from E8, and is then expressed in the forebrain, hindbrain, and spinal cord by E10 (Stoykova and Gruss, 1994; Inoue et al., 2000). Mice with homozygous mutation of *Pax6* die upon birth with malformation in the cerebral cortex (Tyas et al., 2003), whereas heterozygous mutation results in development of a thinner cortex, and have small or reduced eyes (Hill et al., 1991; Schmahl et al., 1993; Fukuda et al., 2000; Haubst et al., 2004; Quinn et al., 2007). Mice with cortex-specific KO of *Pax6* have reduced cortical size and an increased volume of the caudal cortex but without affecting thalamocortical identity (Piñon et al., 2008).

Within the telencephalon, *Pax6* is expressed in the VZ of the dorsal telencephalon, as well as the PSB, with a rostral-caudal gradient (Figure 1B; Bishop et al., 2000; Hirata et al., 2002). *Pax6* expression is repressed in the ventral telencephalon by OLIG2, which ensures the ventral identity of the forebrain (Lim et al., 2019). PAX6 promotes the expression of *Ngn2* in the dorsal telencephalon, together specifying dorsal identity (Scardigli et al., 2003). This pattern of expression is largely complimentary to that of genes specifying ventral identity such as *Ascl1, Dlx1/2*, and *Gsx2*. The only overlapping areas in which these genes are co-expressed are the PSB and the VZ of the LGE (Puelles et al., 1999; Cocas et al., 2011). *Pax6* is necessary for specification of the PSB, maintaining the physical boundary as well as a genetic boundary separating the dorsal and ventral telencephalon (Stenman et al., 2003). *Pax6* homozygous mutants fail to develop such a boundary, with upregulation of ventral genes such as *Gsx2*, and downregulation of dorsal genes like *Ngn2* in the dorsal telencephalon (Stoykova et al., 1996, 2000; Toresson et al., 2000; Yun et al., 2001; Quinn et al., 2007). As a result, the ventral telencephalon, in particular the dLGE, is expanded into the dorsal telencephalon, crossing over the PSB. During the course of development, *Gsx2* expressing progenitor cells in the dorsal LGE can change fate by expressing *Pax6*, distinguishing either *Pax6*-expressing (dorsal) and *Gsx2*-expressing (ventral) progenitor cells at the PSB (Cocas et al., 2011). *Pax6* may regulate the formation of this boundary *via* regulation of cell adhesion molecules (Tyas et al., 2003). Progenitor cells in dorsal and ventral telencephalon expresses R-cadherin and cadherin-6, respectively (Matsunami and Takeichi, 1995; Inoue et al., 1997). Absence of PAX6 in the dorsal telencephalon reduces the expression of R-cadherin, allowing the dorsal and ventral cells to aggregate more readily and consequently disrupts the PSB (Stoykova et al., 1997).

Thalamic patterning is also partly regulated by PAX6 within the dorsal thalamus, where *Pax6* homozygous mutants display altered expression of factors that dictate dorsal identity patterning (Pratt et al., 2000). The patterning function of PAX6 is *via* regulation of *neurogenin2* (*Ngn2*), a bHLH transcription factor (Wang et al., 2011a). NGN1/2 are required for maintaining the normal population of basal progenitor cells, and in *Pax6* homozygous mutants, there is a reduction in *Ngn2* expression, and subsequently reduced a number of basal progenitor cells (Wang et al., 2011a).

Disruption of the PSB also affects the tangential migration of GABAergic interneurons. The PSB functions to limit the number of interneurons arriving at the neocortex, with increased interneurons observed in the neocortex of *Pax6* homozygous mutants along with a loss of PSB (Neyt et al., 1997; Chapouton et al., 1999). Although this outcome may be due to impaired tangential migration, it could also result from ventralization of progenitor cells (Kroll and O’Leary, 2005; Quinn et al., 2007). In these mutants, ventral GABAergic interneuron markers *Dlx1/2, Ascl1, Gsx2*, and *Gad1* are expressed dorsally, and promote differentiation of GABAergic interneurons instead of glutamatergic neurons in cortical progenitors (Kroll and O’Leary, 2005; Long et al., 2009; Wang et al., 2013). This also suggests that *Pax6* represses these dorsal specifying transcription factors, in order to promote the generation of cortical glutamatergic neurons. Unlike early corticogenesis (E12.5), in late corticogenesis (E15.5) there is an addition of differentiated neurons acquiring a GABAergic interneuron phenotype (Schuurmans et al., 2004). These results suggest that *Pax6* controls the differentiation of glutamatergic neurons, whilst suppressing GABAergic interneuron production in late corticogenesis. However, in the diencephalon, *Pax6* also promotes the development of GABAergic interneurons (Robertshaw et al., 2013).

Furthermore, *Pax6* controls the balance between NPCs proliferation and differentiation through regulation of the cell cycle (Manuel and Price, 2005; Georgala et al., 2011). *Pax6* directly regulates various genes that promote neurogenesis, and represses genes essential for non-neuronal fates depending on the histone modifications at the target promoters (Table 3; Sun et al., 2015; Thakurela et al., 2016). *Pax6* homozygous mutants have shortened cell cycles at the start of corticogenesis, but as corticogenesis progresses cell cycle length increases (Estivill-Torrus et al., 2002; Mi et al., 2013). This phenomenon was observed in the cells with the longest cell cycles in the wildtype; in these cells in the *Pax6* mutant mice the shortening of the cell cycle was associated with increased neuronal differentiation (Sansom et al., 2009; Mi et al., 2013; Walcher et al., 2013). Overexpressing *Pax6* increased differentiation of cortical neural stem cells into basal progenitor cells (Sansom et al., 2009). As a result, neural stem cell proliferation is disrupted, and the quantity of neurons is also reduced (Heins et al., 2002; Jones et al., 2002; Hack et al., 2004; Georgala et al., 2011). Hence, an optimal level of *Pax6* expression is necessary for the normal growth and development of the cortex. Further evidence indicates that the balance between proliferation and differentiation is *Foxg1* dependent; *Foxg1* determines whether *Pax6* promotes proliferation or differentiation (Quintana-Urzainqui et al., 2018). *Pax6* itself is regulated by the lncRNA *PAUPAR* in human embryonic stem cells, and such regulation is necessary for cortical differentiation (Xu et al., 2021).

Paired box 6 mutation in humans can result in neurological diseases, more commonly as a result of heterozygous mutations, including intellectual disability, autism, and impaired audition (Malandrini et al., 2001; Davis et al., 2008), likely to be related to reduced cerebral cortex size (Sisodiya et al., 2001; Ellison-Wright et al., 2004). Conversely, only four patients were reported to have mutations in both *PAX6* alleles, of which two survived postnatally (Glaser et al., 1994; Schmidt-Sidor et al., 2009; Solomon et al., 2009). All cases exhibited cerebral cortical malformation, and in the two cases that died before birth, the cerebral cortex was only one-third the size of a normally developed cerebral cortex (Schmidt-Sidor et al., 2009; Table 3).

### Pit-Oct-Unc Class 3 Homeobox 2 Gene

The POU (*Pit-1*, *Oct-1*/*2*, and *Unc-86*) gene family encodes a transcription factor family (Pou1f-Pou6f) of which *Pou3f2* (*Brn2*) encodes a neural transcription factor that is necessary for mammalian CNS development and also for the production of corticotropin-releasing hormone (McEvilly et al., 2002; Castro et al., 2006). *Pou3f2* regulates neuronal differentiation, migration, and upper cortical layer formation during mammalian embryogenesis (McEvilly et al., 2002; Sugitani et al., 2002; Castro et al., 2006; Dominguez et al., 2012; Chen et al., 2018). The protein contains a conserved POU domain composed of 150–160 amino acids, shared by the mammalian transcription factors *P*ituitary-specific PIT1, *O*ctamer transcription factor proteins OCT1/2, and the nematode neural transcription factor *U*NC-86 (He et al., 1989; Ryan et al., 1997; Figure 2A). The DNA binding region of the POU protein is composed of two elements, a POU domain of approximately 75 amino acids present near the N-terminal and a classical HD of 60 amino acids located near the C-terminal separated by a short linker sequence (Figure 2A; Sumiyama et al., 1996; Ryan et al., 1997). Both domains are comprised of a helix-turn-helix structure (4 alpha helices in the POU domain and 3 alpha helices in the HD), which enables DNA recognition and confers DNA-binding specificity at the third helix (Klemm et al., 1994; Cook et al., 2008).

Interactions between the POU domain and its target sequence occur by recognition followed by specific binding to the canonical ATGCAAAT octameric sequence (Figure 2B). However, the linker region between the POU domain and HD is flexible (Herr and Cleary, 1995). The POU3F linker can fold as an alpha-helix which allows homo- or heterodimerization with the target DNA sequence (Blaud et al., 2004). POU3F2 has been reported to form homodimers on an octamer-like sequence of the L-amino acid decarboxylase (AADC), corticotropin (CRRH) and aldose C gene promoters in a non-cooperative fashion (Blaud et al., 2004). *Pou3f2* is located on human chromosome 6q16.1 and dysregulation of this gene has been reported in disorders such as schizophrenia and bipolar disorder, as well as in melanoma (Table 3; Goodall et al., 2004a; Simmons et al., 2017; Chen et al., 2018; Ding et al., 2021).

The onset expression of *Pou3f2* occurs in the VZ of the whole cortical lateral-to-medial axis during early brain development and in the paraventricular nuclei (PVN) of the hypothalamus (Figure 1B; He et al., 1989; Nakai et al., 1995; Dominguez et al., 2012). Using an antibody that detects both BRN1 and BRN2, POU3F2 expression was detected in radial migrating cells from the VZ up to the superficial cortical layers at P0 in mouse brain (Dominguez et al., 2012). Embryonic mice with homozygous *Pou3f2* mutations exhibit hypothalamic and pituitary deficiencies, such as hypoplastic posterior lobe of the pituitary gland and failure to express corticotropin-releasing hormone in the PVN, and die soon after birth (Nakai et al., 1995; Schonemann et al., 1995). *Pou3f2/3* (*Brn1/2*) DKO mice display an abnormal brain phenotype with decreased neocortical thickness and significant reduction of upper layer cells (Sugitani et al., 2002). The olfactory bulb is hypoplastic, the cerebellum is less foliated, accompanied by loosely packed Purkinje cells. Therefore, failure of radial migration results in cortical laminar inversion in the mutant mice (McEvilly et al., 2002; Sugitani et al., 2002). Therefore, *Pou3f2/3* transcription factors redundantly regulate cortical neuron migration and therefore layer production, in addition to neuronal differentiation (Castro et al., 2006).

Two potential mechanisms have been suggested to explain the disruption in the cortical layering defect: *via Pou3f2/3* regulation of CDK5 regulatory subunits *p35* and *p39* in migrating neurons (McEvilly et al., 2002) or through *Pou3f2/3* regulation of *Dab1* (Sugitani et al., 2002). The *Pou3f2/3* double mutant display similar phenotypic abnormalities (McEvilly et al., 2002) to Cdk5-null mutants and *p35/p39*-null mutants (Ko et al., 2001). However, *Pou3f2/3* expression is observed in both a late pool of neural precursor cells as well as in postmitotic neurons, including *Tbr1*+ cells in the cortical plate (Dominguez et al., 2012). Interestingly, when *Pou3f2* is downregulated, there is an excessive number *of Tbr1*+/*NeuroD1*+ cells accumulating within the IZ (Dominguez et al., 2012). POUF3F2 may also regulate *Dab1* as the loss of *Dab1*+ cells in neurons was observed at a later phase (McEvilly et al., 2002; Sugitani et al., 2002; Dominguez et al., 2012). Furthermore, *Dab1* expression was markedly reduced in the *Pou3f2/3* DKO mice at a late stage during which neurons fail to reach the marginal zone and remain beneath the cortical subplate (Sugitani et al., 2002).

Pit-Oct-Unc class 3 homeobox 2 interacts co-operatively with other transcription factors to regulate a number of neurodevelopmental genes, including *Ascl1* in the regulation of Notch signaling, thereby controlling cell cycle exit of progenitors in addition to neuronal differentiation and radial migration in the embryonic telencephalon (Artavanis-Tsakonas et al., 1999). Disruption of POU3F2 binding was shown to prevent transcription of Notch pathway target genes, *Delta1* and *Hes5-1* (Castro et al., 2006). In contrast, overexpression of *Pou3f2* and *Ascl1* in chick neural tube resulted in excessive migration of electroporated cells in the marginal zone of the neural tube and disrupted neuronal differentiation (Castro et al., 2006).

*Pit-Oct-Unc class 3 homeobox 2* dysregulation can have severe neurodevelopmental impacts, contributing to brain malformation, neurodevelopmental delays, and neuropsychiatric disorders (Table 3; Castro et al., 2006; Chen et al., 2018; Hashizume et al., 2018; Westphal et al., 2018; Ding et al., 2021). *POU3f2* has been found to be associated with schizophrenia and bipolar disorder, as a hub for a gene regulatory network related to these disorders (Potkin et al., 2009; Mühleisen et al., 2014; Chen et al., 2018; Pearl et al., 2019; Ding et al., 2021). When POU3F2 is overexpressed in NSCs, several genes which are differentially expressed in the prefrontal cortex of people suffering from schizophrenia and bipolar disorder, are dysregulated. This confirms the role of POU3F2 as a key regulator of gene expression in these disorder (Pearl et al., 2019). POU3F2 and PAX6 were found to regulate the transcription of *TRIM8* (Ding et al., 2021) as well as the *VRK2* (Pearl et al., 2019), other genes associated with schizophrenia and bipolar disorder (Gandal et al., 2018; Li et al., 2018; Ding et al., 2021). While POU3F2 is crucial in regulating genes involved in CNS development, it is also a lineage-determining transcription factor crucial for the regulation of melanocytic lineage. It is overexpressed in many cancer types including carcinomas, neuroblastomas, and melanomas (Schreiber et al., 1990, 1992; Thomson et al., 1995; Leonard and Bell, 1997). Upregulation of *POU3F2* represses Microphthalmia-associated transcription factor (MITF) expression in some melanomas by binding to its promoter region, which drives the cells to adopt a more stem-like and aggressive phenotype (Goodall et al., 2004a; Bonvin et al., 2012). This upregulation is due to the activation of BRAF, a key component of the mitogen-activated protein (MAP) kinase signaling pathway (Goodall et al., 2004b).

### Non-cell Autonomous and Combinatorial Roles of Homeodomain-Containing Transcription Factors

Other than the regulatory functions discussed above, homeodomain-containing transcription factors can regulate forebrain development through *non-cell autonomous* roles as well as by combinatorial modes of action. As an example, PAX6 exhibits non-cell autonomous activity in the development of other organs such as the eye and spinal cord (Collinson et al., 2004; Lesaffre et al., 2007; Di Lullo et al., 2011). This activity is due to two short sequences found within the HD, which are considered essential for secretion and internalization (Prochiantz and Joliot, 2003; Joliot and Prochiantz, 2004). This suggests that some homeobox genes encode transcription factors that have the ability to act as signaling molecules, and are capable of intercellular transfer. Disruption of extracellular PAX6 has functional consequences, leading to defective eye development, with reduction in eye size (Lesaffre et al., 2007). PAX6 extracellular activities can affect cell migration in the embryonic chick spinal cord (Di Lullo et al., 2011). OPCs, a highly migratory cell population, were studied, also due to their delayed specification and dorsal shift in *Pax6* mutants (Sun et al., 1998). OPCs were observed to be in close proximity to PAX6+ cells, and the ablation of extracellular PAX6 resulted in reduced migration of the OPC population (Di Lullo et al., 2011). Another transcription factor shown to have non-cell autonomous activity is OTX2 in the visual cortex, during the regulation of the timing of heightened plasticity, an important timepoint for proper visual development (Lee et al., 2017; Apulei et al., 2018). Extracellular OTX2 has been shown to regulate expression of *Gadd45b*, a gene that may play a role in epigenetic gene activation, as a downstream target for modulating visual cortex plasticity (Ma et al., 2009; Apulei et al., 2018). These examples demonstrate that homeobox genes, through their encoded transcription factors can also function non-cell autonomously. However, this role is yet to be fully understood for the majority of homeobox genes.

In addition, although transcription factors display highly specific expression patterns, many are co-expressed at early stages of development, and work in a combinatorial manner. This concept of a *combinatorial code* has been well documented and studied in the spinal cord (Sugimori et al., 2007; Sagner and Briscoe, 2019). The organization and patterning of the spinal cord is initiated during the development of the neural tube, in accordance with the activities of various morphogens that induce different transcription factor families (Briscoe et al., 2000; Jessell, 2000). A similar code is under active study for forebrain development, where cortical regionalization and patterning are tightly regulated by a transcriptional network consisting of transcription factors and their regulatory elements (Ypsilanti et al., 2021). In particular, cortical expression of transcriptional network members at E11.5 in the mouse forebrain is either in a gradient or in homogenous patterns, with coregulation by transcription factors such as *Pax6*, *Emx2*, and *Nr2f1* (Muzio et al., 2002b; Ypsilanti et al., 2021). Also, transcription factors expressed in the pallium have been implicated in co-binding to regulatory elements through chromatin conformation analysis (Ypsilanti et al., 2021). Future directions employing co-immunoprecipitation, ATACseq, ChIPseq and ChIP-re-ChIP experiments will enable increased understanding of how transcription factors work cooperatively in the regulation of forebrain patterning and regionalization.

## Conclusion

In this review of vertebrate forebrain development (Figure 1), selected transcription factors from the HD, paired, POU and TALE HD gene families necessary for forebrain development have been discussed and summarized (Table 1 and Figure 2). Where known, gene targets of these transcription factors have been specified (Table 2) and correlations to human diseases, including neurodevelopmental disorders and brain tumors have been briefly outlined (Table 3). Further studies are necessary to delineate protein-protein interactions and to identify and characterize post-translational modifications, such as phosphorylation, sumoylation, and ubiquitination, which regulate transcription factor function and link these modifications to signaling pathways in CNS development and disease.

## Author Contributions

RL, AG, ER, MF, JW, and DE conceived and wrote the manuscript. RL, MF, and AG designed and generated the figures. All authors contributed to editing and approved the final version of the manuscript.

## Conflict of Interest

The authors declare that the research was conducted in the absence of any commercial or financial relationships that could be construed as a potential conflict of interest.

## Publisher’s Note

All claims expressed in this article are solely those of the authors and do not necessarily represent those of their affiliated organizations, or those of the publisher, the editors and the reviewers. Any product that may be evaluated in this article, or claim that may be made by its manufacturer, is not guaranteed or endorsed by the publisher.
